# Patients’ Experiences in the Transition From Hospital to Home Palliative Care: A Systematic Review and Thematic Synthesis of Qualitative Studies

**DOI:** 10.1177/23779608251334031

**Published:** 2025-04-15

**Authors:** Sara Cruz, Carla Fernandes, Bruno Magalhães

**Affiliations:** 1 Institute of Biomedical Sciences Abel Salazar (ICBAS), University of Porto, Porto, Portugal; 2 Department of Surgical Oncology of the Portuguese, Institute of Oncology of Porto, IPO-Porto, Portugal; 3 Research Unit in Oncology Nursing IPO Porto Research Center (CI-IPOP), Portuguese Oncology Institute of Porto (IPO Porto) / Porto Comprehensive Cancer Centre (Porto.CCC) &RISE@CI-IPOP (Health Research Network), Porto, Portugal; 4 Nursing School of Porto, Porto, Portugal; 5 CINTESIS@RISE, Nursing Scholl of Porto (ESEP), Porto, Portugal; 6 School of Health, Department of Nursing, University of Trás-os-Montes and Alto Douro (UTAD), Vila Real, Portugal; 7 RISE-Health – Health Research Network, Faculty of Medicine, University of Porto, Porto, Portugal; 8 Clinical Academic Centre of Trás-os-Montes and Alto Douro (CACTMAD), Vila Real, Portugal

**Keywords:** hospices, palliative care, patient experience, qualitative research, systematic review, terminal illness, thematic analysis

## Abstract

**Introduction:**

The concept of transition refers to the shift from hospital-based care to home-based palliative care, encompassing the physical, emotional, and logistical adjustments patients and families face. This study aimed to synthesize the experiences of people in palliative situations at home.

**Methods:**

A systematic review using thematic synthesis was guided using Preferred Reporting Items for Systematic Reviews and Meta-Analyzes (PRISMA) to organize the extracted information. Preparation of the qualitative synthesis followed ENTREQ—*Enhancing transparency in reporting the synthesis of qualitative research* recommendations. The literature search was carried out in MEDLINE, CINAHL, Psychology and Behavioral Sciences Collection, ProQuest, and Worldcat, until October 31, 2023, for articles addressing the experiences of people over 18 years of age in a palliative situation at home. Data analysis employed thematic synthesis, involving inductive coding, development of themes, and interpretative synthesis to provide a comprehensive understanding of patient experiences.

**Results:**

Of the 441 articles identified, 17 studies were included. Data analysis was guided by Meleis ‘s Theory of Transitions, and six distinct categories were included in the conditions of the transition (facilitators or inhibitors): “Personal Facilitators,” “Community Facilitators,” “Social Facilitators,” “Personal Inhibitors,” “Community Inhibitors,” and “Social Inhibitors.”

**Conclusions:**

Findings indicate that the unique nature of the palliative condition and self-perception requires nursing care adapted to the person's experiences. The data collected and the analysis carried out in this thematic synthesis of the literature collectively contributed to identifying the facilitating and inhibiting factors regarding the complex transition process, considering the Theory of Transitions. The findings highlight the importance of personalized care approaches that address patients’ emotional, social, and logistical needs during the transition to home-based palliative care. They underscore the need for enhanced communication, caregiver support, and accessible healthcare resources to improve patient and family experiences, guiding future interventions and policy development in palliative care.

## Introduction

Globally, palliative care refers to the holistic provision of person-centered care to individuals with life-limiting conditions and their family caregivers across various settings. Delivered by interdisciplinary teams, PC encompasses a range of services aimed at meeting the holistic needs of patients and their caregivers, whether in hospital, community, or home settings (WHO, 2022).

At the end of life, patients experience multiple transitions in care, with the transition from hospital to home being pivotal in ensuring that the wishes of the place of death are upheld. Understanding this transition is essential to improving palliative care experiences and outcomes.

The end of life can be described as a unique experience in which each person has individual needs and desires to face death calmly (Radbruch et al., 2020).

Home-based palliative care has gained prominence as many patients express a desire to remain at home during their end-of-life journey. This model provides an environment that fosters comfort, dignity, and family connection, but also presents unique challenges, including the coordination of care, the emotional burden on family caregivers, and the logistical demands of managing medical needs in a non-clinical setting. Despite its benefits, the delivery and outcomes of home-based PC vary across contexts, influenced by healthcare infrastructure, social support, and resource availability (Josefsson et al., 2018).

Specialized palliative care in home settings can benefit the person in a palliative situation and caregivers in different settings and in many countries. Even with professional support, home care can result in substantial emotional, social, and physical demands on informal caregivers (Radbruch et al., 2020). These demands increase in resource-poor contexts, where there is little or no professional support for informal home care (Salifu et al., 2021).

The concept of transition is pivotal in healthcare education and practice, often describing changes in health status, roles, or expectations. In nursing education, it is used to describe people's changes in health status, role relationships, or expectations (Meleis et al., 2000). In this study, the concept of transition specifically refers to the process through which patients adapt to receiving palliative care at home. This includes not only the physical transition from hospital to home but also the emotional, social, and logistical adjustments required to navigate this phase of care (Meleis, 2010). The theoretical framework by Meleis et al. (2000) was chosen for its established utility in understanding transitions in health and illness, particularly in nursing. This framework provides a structured approach to analyzing the complex changes experienced by patients in palliative care at home. Home refers specifically to the patient's personal residence, excluding long-term care facilities or institutional settings.

Finding the best way to support people with advanced illnesses as they experience transitions inevitably requires a qualitative approach to data. To date, qualitative studies have mainly contributed to understanding what it is like to live with incurable illnesses and a specific type of transition (e.g., from hospital to home), but they have not focused on the experiences of transitions during later stages from the perspective of the person experiencing the palliative situation in the various types of transitions in care environments. The current study seeks to fill this gap and contribute to new knowledge, focusing on those with advanced illness at the end of their lives, to shed light on people's experiences in the home.

This study aims to address this gap by conducting a systematic review and thematic synthesis of qualitative studies on the experiences of adult patients in home palliative care. Guided by Transitions Theory, this research aims to provide insights that inform care practices, enhance patient and caregiver support, and contribute to the development of contextually relevant policies.

By synthesizing existing evidence, the review seeks to provide insights into the challenges and facilitators of this care model, guiding improvements in practice, policy, and research.

## Methods

This study aimed to synthesize the lived experiences of adult patients receiving home-based palliative care using a systematic review and thematic synthesis of qualitative studies. This research sought to explore how patients navigate the transition to home care, the challenges they face, and the factors that enhance or hinder their quality of life during this phase.

1. What are the lived experiences of adult patients in home-based palliative care?
2. How do patients perceive and navigate the transition from hospital to home-based palliative care?
3. What challenges and facilitators influence the emotional, social, and physical well-being of patients in this context?
4. What implications do these experiences have for improving care practices, policy development, and future research?

For the purposes of this review, palliative conditions were defined as chronic, life-limiting illnesses requiring multidisciplinary symptom management and holistic care. This includes, but is not limited to, advanced cancer, progressive neurological disorders (e.g., amyotrophic lateral sclerosis, multiple sclerosis), end-stage organ diseases (e.g., heart failure, chronic obstructive pulmonary disease), and other terminal conditions as outlined in WHO (2022) and associated guidelines.

### Design

A systematic review using thematic synthesis (Thomas & Harden, 2008) was guided using Preferred Reporting Items for Systematic Reviews and Meta-Analyzes (PRISMA) (Moher et al., 2009) to organize the extracted information. Preparation of the qualitative synthesis followed ENTREQ—Enhancing transparency in reporting the synthesis of qualitative research recommendations (Tong et al., 2012).

This study adhered to the principles of a systematic review, focusing on qualitative studies that specifically explored the experiences of patients receiving home-based palliative care. The limited number of included studies reflects the application of strict inclusion criteria to ensure methodological rigor and relevance. While this review does not aim to comprehensively map the breadth of literature on this topic, typically the goal of a scoping review, it provides an in-depth synthesis of studies that meet the specified criteria, consistent with systematic review methodology.

### Search Methods

The research question was defined according to the acronym PCC (Population, Context, and Concept) in accordance with the inclusion and exclusion criteria presented in Table 1. This study sought to investigate the experiences of people in palliative situations, so inclusion criteria were established for studies with qualitative methods in data collection (e.g., focus groups and individual interviews) and qualitative methods in data analysis (e.g., phenomenology and grounded theory), one mixed-methods study was included in this review, as its qualitative component directly addressed the research questions and met the inclusion criteria. Exclusion criteria included a population under 18 years old and a population not receiving palliative care at home.

**Table 1.** Definition of Inclusion Criteria.

*Inclusion Criteria*	
*Population (P)*	Adult palliative patients
*Concept (C)*	Studies that address the experiences of adult palliative patients
*Context (C)*	Studies in which participants are solely and exclusively in home care, home
*Types of studies*	Qualitative methods in data collection (e.g., focus groups and individual interviews) and qualitative methods in data analysis (e.g., phenomenology and grounded theory)
*Language*	Studies published in any language
*Publication date*	No time limit, but documents collected by October 31, 2023

The respective descriptors identified in English, using search syntaxes adapted to each database, were used to carry out the search (Table 1—Research Question): MEDLINE^®^ (Medical Literature Analysis and Retrieval System Online), CINAHL^®^ (Cumulative Index to Nursing and Allied Health Literature), Psychology and Behavioral Sciences Collection, ProQuest, and Worldcat.

Combinations of medical subject descriptors/headings (MeSH), subject headings, and subject terms were applied to each database using the Boolean operators ‘OR’, ‘AND’, and ‘*’, which improved the search by allowing word variation. The first author conducted the research without any language restrictions or time limit but collected until October 31, 2023, as presented in the annex in Table 1 (Research Question).

### Article Selection

**Figure 1.**
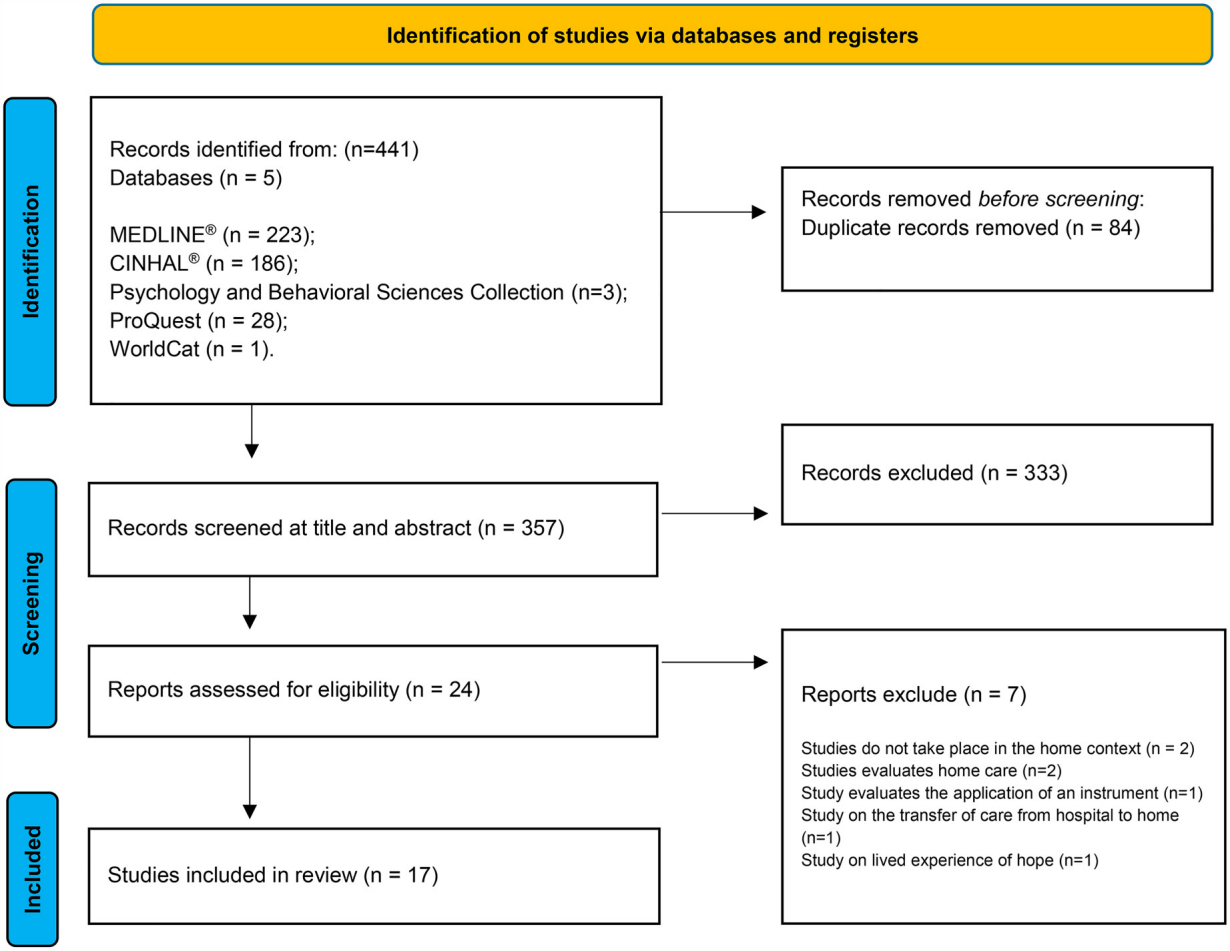
PRISMA Flow Diagram.

Each search result was entered into reference management software (Endnote X8^®^; https://endnote.com, Philadelphia, United States of America). Duplicate references were removed, and the initial selection by title and abstract was carried out independently by two investigators (SC and CF) according to the defined inclusion and exclusion criteria. The full texts of the remaining references were retrieved for reading to decide on article inclusion/exclusion. A third investigator (BM) was invited to reach consensus where necessary to determine whether to include or exclude an article. The PRISMA was used to organize the information extracted from the articles (Figure 1).

### Quality Appraisal and Data Extraction

Firstly, the data extraction and synthesis process included complete and successive readings of the articles, from which data were extracted independently. Then, all researchers re-evaluated the extracted data, reviewed the initial cases with disagreement, and reached a consensus. One author (SC) extracted and tabulated information from the included studies. The articles that made up the reference sample were coded (Sn) and numbered in ascending order from oldest to most recent.

Assessors (CF, BM) independently assessed the quality of the included papers, using the Critical Appraisal Skills Program (CASP) for qualitative research (https://casp-uk.net/checklists/casp-qualitative-studies-checklist.pdf). The CASP instrument was applied independently by two reviewers (SC, CF). A third reviewer (BM) was invited to reach a consensus if necessary. Articles were scored for each criterion with 1—if the criterion was met, 0—if the criterion was not met, and 0.5—if the criterion was partially met (Notley et al., 2015). Since the instrument consists of ten items, the maximum score for an article would be 10 points. The risk of bias was assessed using CASP, and studies with a score less than or equal to five points were not considered.

Data from the studies were extracted using specific extraction forms. The following information was recorded for each study: a) authorship, year of publication, and country; b) objective of the study; c) methodological design d) characteristics of the participants; e) data collection method and results, as presented in Table 2.

**Table 2.** Characteristics of the Selected Studies.

Study ID	Author, Year Country	Aim of the Study	Type of Study	Participants	Data Collection Analyses	Findings (Data were Categorized into Main Themes)
S1	(20) Sweden	To obtain an understanding of patients’ experiences of palliative care at home with service from district nurses	Phenomenological	9 patients with cancer (3 women, and 3 men) Ranged in age from 54 to 85 years (median 79.5 years, mean 73.3 years)	Semi-structured interview Giorgi method analysis	Five subthemes were identified: safe but unsafe at home, a sense of powerlessness, change of everyday life, hope and belief in the futuremand One major theme illustrating the essential meaning “uncertain safety” was identified from the experience with palliative care in the home.
S2	(21) Japan	To identify the experiences of in-home care patients living with progressive neuromuscular illnesses during long course	Phenomenological	9 patients with neuromuscular illness and their families	Semi-structured interview Qualitative induction analysis	Six domains with 12 categories were identified: psychological distress, recipients’ illness experiences from a long-term perspective, changes in the perception of the disease, suffering in living with a difficult disease, support for living with a difficult disease and shaking thoughts during the course of long-term treatment.
S3	Artsanthia, Mawn (22) Asia	To explore the palliative care needs for Thai people living with end-stage renal disease (ESRD)	Phenomenological Mixed method	30 participants with ESRD, 39 family members, four community leaders, and four healthcare providers	Semi-structured interview Interpretive description method The participants completed the revised Edmonton Symptom Assessment Scale, which provided descriptive data on nine symptoms commonly experienced among palliative care patients. In addition, focus group and in person interviews were conducted with study participants	Four main themes arose from the mixed-methods data: tremendous suffering, economic consequences, inadequate community support, and concern for the future.
S4	(23) Sweden	To explore the meanings of living with eating deficiencies at the end of life among people admitted to specialist palliative home care	Phenomenological	12 persons, with various diagnoses and eating deficiencies, admitted to two specialist palliative home care units, participated	Semi-structured interview Interpretive description method	Three meanings of eating deficiencies that involve a multitude of existential and psychosocial challenges related to illness, bodily decline, and awareness of impending death: (1) resisting death by eating, (2) struggling with social gatherings around food, and (3) eating to please and unburden others.
S5	(24) Japan	To investigate the end-of-life wishes and decision-making among Japanese elderly people who required home care services	Phenomenological	102 elderly people (47 males, 55 females)	Semi-structured interview Kawakita Jiro method analysis	Five themes were extracted: anxiety about the future, abandonment of control, clinging to current daily life, precarious mutual support and delegating decision-making.
S6	(25) South America (Colombia)	To describe the experiences of the patient-family caregiver dyad in palliative care during the transition process from hospital to home	Phenomenological	12 patient-family caregiver dyads participated in the study (12 patients and 12 caregivers)	Semi-structured interview Data were stored in ATLAS-ti software Interpretive description method	Seven themes that describe the experience of the individual in palliative care and their family caregiver during the transition from hospital to home care: life changes, emotional and spiritual burden, caring actions, physical discomfort and pain, coping, bond between the person receiving palliative care and their caregiver and expected responses.
S7	(26) Bosnia-Herzegovina (Europe)	To explore the perceptions, experiences, and expectations of terminally ill patients, regarding their health care and social support	Phenomenological	62 purposely selected patients	Semi-structured interview Both qualitative and quantitative analyses were conducted, using an inductive thematic approach	Five needs emerge from the quantitative analysis of the ratings given by the interviewed patients show in decreasing order the relative importance of lack of autonomy in daily activities, depression and loneliness, pain control and symptom management, burden on families, deontology and professionalism and finally the lack of information.
S8	(27) Sweden (Europe )	To explore patients in need of palliative care despite diagnosis, and their relatives’ experiences receiving advanced home care	Phenomenological	11 interviews were conducted: 8 with patients and 1 or 2 close relatives together; and 3 with the patient alone	Unstructured in-depth interviews Qualitative content analysis	Three main categories: create a safe environment, see the person, and better to manage care at home.
S9	(28) German (Europe)	To determine the key components contributing to a sense of security and how they relate to each other as experienced by patients and family caregivers in specialist and generalist palliative home care	Phenomenological	197 patients and 10 carers completed interviews	Semi-structured interview Deductive qualitative content analysis with the factor sense of security as primary theory-based concept	Nine subcategories that were all mentioned more frequently by specialist than generalist palliative home care recipients in the following order of priority and relation: patient-centeredness: availability, provision of information/education, professional competence, patient empowerment, and trust; organizational work: comprehensive responsibility, external collaboration, and internal cooperation, and direct communication.
S10	(29) United Kingdom (Northern England and Northern Scotland)	To explore the palliative care needs of patients living with severe COPD and their caregivers, (2) to understand views of accessing and providing palliative care and factors influencing these experiences, and to explore to what extent palliative care and COPD services have been integrated	Phenomenological	20 patients, 6 carers, and 25 health professionals	Semi-structured individual interviews and focus groups A framework matrix also was generated summarizing the data by category and theme from each transcript (NVivo 12 software)	Four themes were generated from interviews: management of exacerbations, palliative care needs, access to palliative care and pathways, and integration of palliative care support.
S11	(30) Toronto, Canada	To explore patients’ and caregivers’ expectations and subsequent experiences of the hospital-to-home transition while receiving palliative care, and build a substantive grounded theory to enhance the understanding of hospital-to-home transitions from the patient and care- giver perspective	Longitudinal, prospective qualitative study Used grounded theory methodology	39 participants: 18 patients, 7 caregivers, and 7 patient-caregiver dyads participated	Semi-structured interviews Inductive description method (MaxQDA software)	Identified various needs: health and well-being needs, and practical needs (i.e., transportation, setting up the home for care, care providers in the home). Several enablers and disablers modified the likelihood of needs being met (e.g., caregiver role, education on symptom management, uncertainty, financial resources).
S12	(31) China	To explore the needs and experiences of palliative home care among patients with advanced cancer in China	Phenomenological	15 participants were interviewed in this study, diagnosed with stage IV cancer	Semi-structured interviews Thematic analysis (NVivo 12.0)	Five themes related to participants’ needs and experiences of PHC were as follows: physical need; psychological experience; spiritual need; social need; and information need.
S13	(6) Ghana, West African	To explored palliative and end-of-life care experiences of family caregivers and patients living at home in a resource-poor context in Ghana	Phenomenological Social constructivist theory and interpretivism underpin this study where co-creation of knowledge and ideas are the product of the interaction between researcher and participants	23 men with advanced (stage III or V) prostate cancer, 23 family caregivers, and 12 health professionals participated	Semi-structured interviews Thematic analysis (NVivo 12.0)	Three main themes from the data are the practical and emotional issues about care at home, caring in the absence of the support of health staff, and pain management.
S14	(32) Brazil	To explore the meanings and experiences of patients with terminal chronic diseases and their caregivers, who face the imminence of death in the home environment after hospital discharge	Qualitative study used constructivist grounded theory	11 patients and 10 family caregivers participated in the study	Semi-structured interviews Inductive analysis of the data was carried out, following Charmaz's grounded theory analysis method	Three interrelated data categories emerged: floating between acceptance and resistance, perceiving the proximity of death, analyzing the end from other perspectives: it is in the encounter with death that life is understood and accepting the path: between the love of letting go and the love of wanting to stay.
S15	(33) Canada	To understand the experience of patients with advanced colorectal cancer and family caregivers who received early palliative care supports from a specialist palliative care nurse and compared those experiences with participants who experienced standard oncology care prior to implementation of early palliative care	Phenomenological Qualitative and patient-oriented study	7 patients living with advanced colorectal cancer and five family caregivers	Semi-structured telephone interviews with two cohorts of patients with advanced colorectal cancer before and after implementation of an early palliative care pathway Thematic analysis by Braun and Clark (NVivo)	Four main themes shaped their experience of early palliative care: care coordination, perception of palliative care, advance care planning, coping with advanced cancer, and patient and family engagement. These findings were compared with experiences of 15 patients and seven caregivers prior to pathway implementation.
S16	(34) Canada	To explore the lived experience of everyday life for working-aged adults living with advanced cancer, and how these changes over time	A longitudinal hermeneutic phenomenological approach was employed	8 adults living with advanced cancer	Semi-structured interviews Inductive thematic analysis, and findings mapped against the Model of Human Occupation and illness experience literature Colaizzi's data analysis methods were modified to incorporate Saldaña's longitudinal analysis methods (NVivo)	Three themes were constructed from the data: The intentional pursuit of engagement in everyday activities, the challenge of unrelenting change and loss as death approached, adapting to change is an active, ongoing process.
S17	(35) Colombia	To describe the beliefs and health- related cultural practices of adult patients in a palliative home care program in the city of Bogotá	Qualitative ethnographic study	9 patients requiring palliative care	Semi-structured online interviews For data analysis, the guidelines of Leininger's Cultural Care Theory based on the Sunrise Model were considered (NVivo)	Three main categories were identified: cultural practices, beliefs about maintaining poor health and alleviating symptoms, and experiences adapting to illness and death, resulting in 13 subcategories.

### Data Analysis

Firstly, the data extraction and synthesis process included complete and successive readings of the articles, from which data were extracted independently. Then, all researchers re-evaluated the extracted data, reviewed the initial cases with disagreement, and reached a consensus.

The first data analysis stage involved carefully reading and re-reading each study to identify, organize, compare, and evaluate the available data. A thematic data synthesis was carried out (Thomas & Harden, 2008). Based on this approach, a three-step analysis process was carried out (Barnett-Page & Thomas, 2009) as proposed. The first step included line-by-line coding of the text segments present in the results and discussion sections of the included article, displaying the qualitative analysis carried out by each author. ATLAS.ti ^®^ software (Version 23; Scientific Software Development GmbH, Berlin, Germany) was used to code the results of the studies line-by-line. This first coding was carried out by three researchers. The first researcher analyzed all articles (SC), and each of the remaining two researchers (BM, CF) analyzed articles randomly.

All findings were classified according to JBI credibility levels as “unequivocal,” “credible,” or “unsupported” (Aromataris, Munn & editors, 2020). From the classifications of the findings and illustrations, the researchers created categories that, when put together, produced a single comprehensive set of findings. The process of grouping the categories was presented in a descriptive synthesis format (Aromataris et al., 2020). The categories emerged from the assumptions of the Theory of Transitions (Meleis, 2010).

Two independent reviewers (SC, CF) evaluated the studies using the standardized JBI Critical Instrument Appraisal Skills Program Qualitative Research Checklist (Campbell et al., 2020; Lockwood et al., 2020). Any disagreements that arose between reviewers were resolved through discussion or with a third reviewer (BM).

The JBI ConQual process (Aromataris et al., 2020; Lockwood et al., 2020) was followed to establish the confidence of evidence for each synthesized finding. This included rating the research, assessing the dependability of the studies, and the credibility of the findings. Each article in an individual synthesized finding was initially pre-ranked using the JBI ConQual ranking; each article is initially ranked from “High” to “Very Low”; qualitative articles are ranked “High”, while text and opinion articles are classified as “Low” (Munn et al., 2014). The rating of synthesized findings can change or stay the same depending on their dependability and credibility. Dependability assesses whether the study was conducted properly to meet its objectives, using five questions from the JBI critical appraisal scores. Credibility evaluates whether the author's interpretation aligns with the supporting data.

Based on these criteria, findings are rated as: (a) Unequivocal: Supported by clear evidence, leaving no room for doubt; (b) Credible: Supported by some evidence, but open to challenge; or (c) Not supported: Lacking enough evidence to back the findings (Munn et al., 2014). A final ConQual score after dependability and credibility assessment was established for each synthesized finding, as presented in the Supplementary Data—Joanna Briggs Institute Appraisal Tool and Scoring System.

## Results

### Study Selection Results

Preferred flowchart Reporting Items for Systematic Reviews and Meta-Analysis (PRISMA) (Page et al., 2021) in Figure 1 shows that 441 studies were identified through the databases. Eighty-four duplicates were removed, and 357 records were excluded after title and abstract screening. The full texts of 24 studies were screened for eligibility. A total of 17 studies met the inclusion criteria (Figure 1).

### Characteristics of the Studies

The 17 studies identified in the sample took place between 2004 and 2023; a total of 546 people in palliative situations in home care were interviewed through a semi-structured interview held in the person's home.

Different methodological approaches were used: phenomenological (*n* = 12) (Aebischer Perone et al., 2018; Ahmed et al., 2023; Appelin & Berterö, 2004; Brose et al., 2023; Dillen et al., 2021; Fu et al., 2021; Hirakawa et al., 2017; Liu et al., 2021; Rocío et al., 2017; Ushikubo, 2005; Wallin et al., 2015; Wennman et al., 2020), phenomenological with a mixed method where a symptom assessment scale was applied (*n* = 1) (Artsanthia et al., 2011), Grounded Theory (*n* = 3) (Isenberg et al., 2021; Prado et al., 2022; Salifu, Almack & Caswell, 2021) and ethnographic (*n* = 1) (Pérez Sandoval et al., 2023).

The analysis methods used were thematic analysis (*n* = 11) (Aebischer Perone et al., 2018; Ahmed et al., 2023; Appelin & Berterö, 2004; Brose, Willis & Morgan, 2023; Fu et al., 2021; Hirakawa et al., 2017; Isenberg et al., 2021; Liu et al., 2021; Rocío et al., 2017; Salifu et al., 2021; Wennman et al., 2020), Grounded Theory (*n* = 3) (Artsanthia et al., 2011; Pérez Sandoval et al., 2023; Prado et al., 2022), interpretative descriptive method (*n* = 2) (Ushikubo, 2005; Wallin et al., 2015) categorization deductive (*n* = 1) (Dillen et al., 2021) and constant comparative method (*n* = 0). The characteristics of the studies are summarized in Table 2.

### Quality Assessment Results (Dependability/Credibility)

In all studies, the philosophical perspective remained clear: there is congruence between the methodology and the research question or objectives, between the research methodology and the methods used to collect the data, between the research methodology and the representation and analysis of data, between research methodology and interpretation of results. No study presented a statement that situated researchers culturally or theoretically. Three studies (Artsanthia et al., 2011; Fu et al., 2021; Ushikubo, 2005) do not mention the investigator's influence on the research and vice versa, and one study (Aebischer Perone et al., 2018) is unclear in this mention. Regarding the representation of participants and their voices, two studies (Artsanthia et al., 2011; Ushikubo, 2005) are not clear on this representation.

Regarding the statement on the ethics approval process and the relationship of conclusions with the analysis and interpretation of data are present in all studies analyzed.

The studies were of high methodological quality. Overall, their dependability scores ranged from 7 to 9 (Supplementary Data—Joanna Briggs Institute Appraisal Tool and Scoring System).

Unlike the focus of critical appraisal commonly performed as part of the systematic review process, when evaluating the credibility of findings, the focus was not on the entire research enterprise but, more importantly, the results of the authors’ interpretative analysis, more commonly referred to as “findings” in the literature (Sandelowski & Leeman, 2012).

A total of 281 unequivocal and 17 equivocal findings were included and there were no unsupported findings in the included articles. The synthesized findings are considered valid, as the ConQual scores of nine of them are classified as “moderate” and the ConQual scores of eight as “high” (Supplementary Data—Joanna Briggs Institute ConQual Score).

### Experiment Results

Two hundred and eighty-one findings, 34 categories, and 2 synthesized findings were identified from 17 studies. All findings were classified as unequivocal.

In an inductive perspective of data analysis, guided by the Theory of Transitions, two explanatory themes, facilitators and inhibitors, identified by Meleis (Meleis, 2010), allowed us to synthesize the experiences of people in palliative situations at home. The transitions theory by Meleis (Meleis et al., 2000) was chosen to provide a structured framework for understanding the multidimensional changes experienced by patients, including shifts in roles, relationships, and care needs.

While people in a palliative situation at home experience different experiences, some conditions facilitate, and others inhibit adaptation to the situation.

Next, the main category with subcategories will be presented. The six distinct categories were classified as transition conditions (facilitators or inhibitors): “Personal Facilitators,” “Community Facilitators,” “Social Facilitators,” “Personal Inhibitors,” “Community Inhibitors,” and “Social Inhibitors.”

Although not the primary focus of the review, the identification of facilitators and inhibitors provides valuable insights into factors that enhance or hinder patients’ experiences during the transition to home-based palliative care.

**Figure 2.**
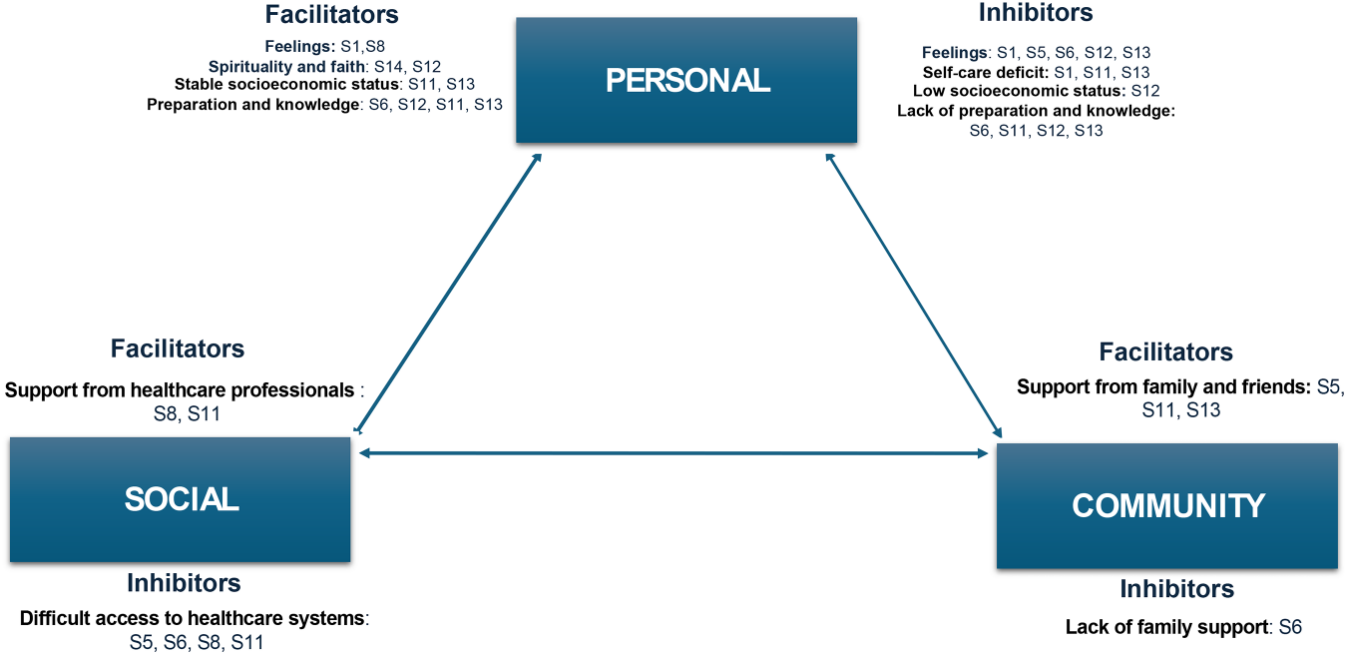
Visual chart of facilitators and inhibitors of the transition of people in palliative care at home.

Based on the qualitative findings, a conceptual model of the lived experiences of people in home palliative care circumstances was derived as a central outcome of this study (Figure 2).

### Facilitating Experiences of People in Palliative Situations at Home

The provision of palliative care at home becomes important when it is considered by the person and their family members to be the most appropriate type of care for the final stage of the person's life in a palliative situation. Recent studies have shown that home palliative care improves patients’ quality of life, increases care delivery, and has a positive economic impact on the healthcare system (Rocío et al., 2017).

In relation to the experience of the person in a palliative situation at home, the content most mentioned by the participants was related to various aspects seen as generally favorable to this process, totaling 74 statements identified as Facilitators. Within this theme, several subthemes were identified in Table 3 and Supplementary Data—Facilitating experiences of people in palliative situations at home.

**Table 3.** An Overview of Themes.

Themes	Subthemes/Denomination for Framework	Example of Supporting Quotes (with Study Number – Sn)	Studies Contributing to the Review Findings
Facilitators	Personal: Feelings	*… but I shall go on fighting … hm … I have a feeling that my life is not finished yet … S1* I *only get reminded of a lot of negative things by visiting the hospital. For me it's just, if I go to the hospital, I have to recover during the following 24 h, it is nothing but negative…it's much better if everything could be managed here (at home). S8*	S1, S8
	Personal: Spirituality and faith	*(PT_BHU_1) I was sad to know, because there are so many things that we would still like to do and cannot. But I have faith, the only one who knows things is God, and only He knows about tomorrow. S14* (FC_PC_3) today we are much more realistic with the situation, it is heading towards the end, we are also going, we are all going to [die] . . . there are some people who are closer by nature, this is her case, for this is clear to me. *S12*	S14, S12
	Personal: Stable socioeconomic status	*I think the commode chair, we had it for 28 days. That will be ending this week, and they want a ridiculous price, like $50 a month to rent. . .we’re going to have to figure out what we’re going to do. (Caregiver 74 M). S11* *We got some herbal medicines from a woman who is known to be an expert in that field. (Eno, Mike's wife). S13*	S11, S13
	Personal: preparation and knowledge	*´When he had the catheter, I would administer the medicine, but a few times he would do it, I help him with the curing procedures, all that, help him to vacate the colostomy and those things I have already learnt how to managé. S6* *C4E1* ‘*I still need to know how to manage those things, I have to pay attention. Although he also pays attention and he knows how his things are applied, the colostomy and all also, he has learnt some things’.C5E5 S12*	S6, S12, S11, S13
	Community: Support from family and friends	*I am grateful that my children, daughter-in-law, and day service center staff kindly take turns caring for me. I want to stay in the house where my husband and I built a life together despite the fact that he was rigid. (Eighty-seven, female, others). S5* “*was happy to be with them and have a chat with them…we get those emotional support from our parish community” (Caregiver 69F). S11*	S5, S11, S13
	Social: Support from healthcare professionals	*And I actually discussed the matter with the doctor here, we sat down and talked about it for almost an hour, about how things would turn out and how it (dying) would be. She said, “We are here for you all the way.” “All the way,” now I know exactly what they mean. S8* *Well, a lot of those ambulance people, they are quite something. Quite organized and better than what they used to be because you’re not doing well if you don’t know when if you’re coming back home you’ve got the voyager chairs . . .stretcher as well as a chair if you’re able to sit. And I thought that was quite interesting because I’ve never been in one before that. I’ll say where’d you get that chair, I can use that at home. (Patient 82F) S11*	S8, S11
Inhibitors	Personal: Feelings	*I have asked once in a while if they can do anything … but they did not answer me really … S1* *I worry that I may not be able to discuss care services with the hospital or care service center if my condition deteriorates. (Seventy-five, male, one-person household)S5*	S1, S5, S6, S12, S13
	Personal: Self-care deficit	*… I want to live as usual but it's not possible … I can’t cook … I can’t go for a walk …” S1* ‘*I am filled with angst, when he says this or that …’ C3E3 S11*	S1, S11, S13
	Personal: Low socioeconomic status	*Over the past three years, I had received more than 20 chemotherapy treatments. The expensive medical bur- den forced us to sell our only house and borrow money from our relatives and friends, racking up medical bills of more than 1 million RMB (Participant 6). S12* *I lived in a rural area, and the rural new cooperative medical scheme could reimburse only 30%. The reimbursement rate was too low to maintain anticancer therapy (Participant 9). S12*	S12
	Personal: lack of preparation and knowledge	*My wife replaced a colostomy bag for me yesterday. I found a macerated peristomal site with surrounding excrement. We had to go back to the hospital (Participant 15). S6* “ *I didn’t know anything and I wasn’t being taught any- thing on how to handle my own condition. You know, so it's a little scary” (Patient 77F). S12*	S6, S11, S12, S13
	Community: Lack of family support	*My mother sometimes thinks I scold her because I tell her to be patient, but I tell her: “mom let's not get too anxious”’. C2E2. ‘I think my mother wants to die at home’. C3E3 S6*	S6
	Social: Difficult access to health systems	*‘The oxygen stops a lot, they ordered medications, we were discharged and at this time we have not gotten authorizations for any of them, not even morphine, nothing’. C1E1 S6* *They can’t see the notes . . . it's probably wrong, because they can’t see the patient records . . . unable to solve it with any consultation—or log in, so it's a pure stone-aged action to sit and send a test result and other stuff by fax. So that's too bad. S8*	S5, S6, S8, S11

The personal facilitators category includes the subcategories of feelings, spirituality and faith, stable socioeconomic status, and preparation and knowledge. According to Afaf Meleis's Transitions Theory the personal conditioning factors of transition are the meanings associated with change, and their changes result from cultural beliefs and attitudes, socioeconomic situation, and preparation and knowledge of the person and family (Meleis, 2010).

During the stay at home of the person in a palliative situation, different meanings can be attributed and can trigger positive feelings based on the conditions related to the condition of palliative care at home. In this sense, feelings associated with happiness, gratitude, and tranquility predominated (Appelin & Berterö, 2004).

With regard to cultural beliefs and attitudes, it was observed that faith and spirituality facilitate the transition process as they bring comfort and acceptance of the condition established by the disease, making the person more centered in their care process (Prado et al., 2022).

The stable socioeconomic status facilitated the transition process as some people were able to adapt their home space for greater accessibility for the person (Isenberg et al., 2021).

The guidance provided by the multidisciplinary team and follow-up at home proved to be facilitators of the transition process, helping to acquire skills for (self)care, aiming to promote the health and independence of the person and their family caregiver (Rocío et al., 2017).

The community facilitators category is related to the person's support network. Support from family and social groups (neighbors and friends) were identified as resources available in the community that make it easier to face changes occurring in the family context (Hirakawa et al., 2017).

As the testimonies portray, the family constitutes the person's main support network, helping with basic activities of daily living (BADL), accompanying them to appointments, and contributing financially to health and transportation expenses when they need to move.

The category facilitators social was expressed by the assistance of healthcare institutions proved to be a facilitator for the transition process (Wennman et al., 2020).

Practical needs that arose included, for example, organizing transport and were assured (Isenberg et al., 2021). Table 3 provides a detailed breakdown of the themes and subthemes identified in the synthesis, illustrating the depth and complexity of patients’ experiences. This level of detail aims to ensure transparency and facilitate future research and practice (Table 3).

### Difficult Experiences of People in Palliative Situations at Home

When advanced illness changes routines and the overall experience of days and hours in everyday life, a person's sense of self is also altered. The deterioration associated with disease progression impairs a person's ability to participate in everyday life and has a detrimental effect on a person's sense of self, dignity, and quality of life.

In relation to the experience of the person in a palliative situation at home, the content most mentioned by the participants was related to various aspects seen as generally unfavorable to this process, totaling 63 statements, identified as Inhibitors. Within this theme, it was possible to identify several subthemes in Table 3 and Supplementary Data—Difficult experiences of people in palliative situations at home.

The personal inhibitors category includes the subcategory feelings, self-care deficit, low socioeconomic status, and lack of preparation and knowledge.

During the transition from palliative care at home, the different meanings attributed to the process triggered negative feelings for the person in a palliative situation. The feelings associated with transition-inhibiting conditions related to the person in a palliative situation at home predominated, expressions that demonstrate inexperience, effort, discouragement, anguish, and incapacity (Appelin & Berterö, 2004; Rocío et al., 2017).

Regarding the self-care deficit, attitudes of the person that generate their dependence were observed, and they were associated with an inhibiting condition for the transition (Appelin & Berterö, 2004).

Economic vulnerability made the transition process difficult, being characterized as an inhibiting factor (Liu et al., 2021).

The lack of knowledge and the feeling of lack of preparation highlight the difficulty faced by the person in a palliative situation, but also by family caregivers in the transition of care, as knowledge is one of the most important needs for them, as it allows them the best perception of the transition experienced, revealing the overcoming of challenges in adapting to the new context of being at home (Liu et al., 2021) (Isenberg et al., 2021).

The community inhibitors category was characterized by a feeling of responsibility for care and a lack of family support. When people in palliative situations do not receive support from family members, as reported in the speeches, an overload occurs, causing physical, emotional, and psychological exhaustion (Rocío et al., 2017).

The social inhibitors category, which characterized the difficulties in accessing services evidenced in the speeches, proved to be a barrier to the reorganization of the person in a palliative situation during their stay at home (Rocío et al., 2017). Some participants mentioned that it was notable that various healthcare providers used different documentation systems for records, which they felt had a negative impact (Wennman et al., 2020) (Table 3).

## Discussion

The central objective of this study was to understand the experience of people in palliative situations at home. The interpretation of the results suggests that the experience is mainly guided by the impact of the disease on the person's life, particularly in the physical, psychological, social, and spiritual dimensions; the needs perceived by the person; the strategies they implement to cope with the disease; and the resources they consider available to them.

According to Afaf Meleis’ Transition Theory, the personal conditioning factors of transition are the meanings associated with change, and their changes result from cultural beliefs and attitudes, socioeconomic situation, preparation and knowledge of the person and family (Meleis, 2010).

During the transition, the different meanings attributed to the process trigger positive feelings based on the conditions related to this circumstance. In this sense, feelings associated with well-being, security and tranquility predominated, as illustrated in the quotes.

### Spirituality and Faith

Spirituality and faith emerged as essential coping mechanisms for many patients during the transition to home-based palliative care. Beyond offering comfort, they provided a sense of meaning, resilience, and hope, particularly in moments of uncertainty and vulnerability. This aligns with existing research indicating that spirituality can serve as a psychological resource that enhances adaptation to terminal illness; religious or spiritual beliefs can facilitate the transition when the person is at home, and the impact of spiritual well-being on decision-making is evident. Spirituality is a key component of general well-being and takes on multidimensional and unique functions (Rego et al., 2020). Beliefs promote the construction of perceptions about reality, giving meaning and direction to life (Macintosh, 1994).

### Socioeconomic Status

Patients from lower socioeconomic backgrounds encountered significant barriers to accessing home-based palliative care, including financial constraints, limited availability of professional caregivers, and reduced access to essential medical equipment. These disparities suggest that economic inequalities directly impact the quality and continuity of end-of-life care, reinforcing the need for policy interventions that ensure equitable access to palliative care services. Our findings align with previous studies demonstrating that lower-income patients often experience fragmented palliative care services due to financial constraints and healthcare system inefficiencies, socioeconomic status, the relative position or order of an individual in a hierarchy based on social and economic attributes expressed in differential access to resources, can condition the transition experience (Meleis et al., 2000).

In the reports, it was observed that financial stability made it possible to make changes in the physical environment, as illustrated in some illustrations, facilitating the transition of care for the person in a palliative situation at home, corroborating the results found in another study. The spatial modification of some parts of the residential area is essential to meet the needs of the person and maintain quality of life and safety in everyday life (Quinn et al., 2023).

### Preparation and Knowledge

Patients and caregivers frequently reported feeling unprepared for the transition to home-based palliative care, particularly in managing symptoms, administering medication, and recognizing signs of disease progression. This lack of preparation often led to heightened anxiety and an increased reliance on emergency healthcare services. These findings underscore the need for structured education programs that equip patients and families with the necessary skills to navigate end-of-life care at home effectively. Our findings align with previous research indicating that insufficient knowledge is a significant barrier to effective home-based palliative care. Moreover, the sick person's need for information changes over time and depends mainly on specific events related to the disease. When this information does not exist, people are dominated by subjective perceptions of the disease, and feelings of impotence, helplessness, and frustration develop, as illustrated in the participants’ quotes (Morey et al., 2021).

The people in the studies analyzed reported having individual knowledge that helped them make decisions regarding the search for health care, social support, and symptom management, with knowledge being a resource to deal with their health condition, which ranges from this is in line with that found in another study in which participants found that receiving the right information in the right place at the right time helped them gain a better understanding of their health status and care plan, which would reduce their anxiety and worries (Guo et al., 2022).

### Social Support

Social support emerged as a fundamental element in facilitating a successful transition to home-based palliative care. Beyond practical assistance, such as medication management and personal care, social support provided emotional reassurance and a sense of stability, helping patients cope with uncertainty and loss of independence. This aligns with prior research indicating that strong support networks contribute to reduced stress and improved quality of life in palliative care settings. The fact that all participants identify a family member as a reference, as a pillar that supports them in all decisions throughout this phase of their life, is a positive aspect, realizing that they have brought closer and strengthened ties and have an impact on the person's well-being in a palliative situation, illustrated by the participants in the studies analyzed and which is in line with what was identified in another study (Dobríková et al., 2016).

The community in which the person lives, seeking to be more present and showing support for the person and family, has had a positive impact on the attempt to provide some normality to their life, getting closer to what it was like before the illness, just as illustrates quotes from participants in the studies analyzed.

Our findings are consistent with previous studies demonstrating that social support plays a critical role in end-of-life care*.* Family members often act as advocates in the healthcare setting, and most participants specified a great need for support from family and friends not only in the practical setting, such as arranging transportation or coordinating care, but also emotionally (Guo et al., 2022).

Family support, available information, support in the decision-making process, available resources, and adequate response to effective needs facilitate the transition (Meleis, 2010).

Regarding health professionals, in a study developed on experiences of transition between care environments from the perspective of patients with advanced illness in specialized palliative care and their family caregivers, participants referred to doctors and nurses as an available resource. Doctors provided information and clarification of doubts, as well as support in managing signs and symptoms. Both families and people with advanced illness praised the doctors who provided continuity and got to know them as individuals and the nurses as providers of self-care support. Nurses promote the process of adaptation to the current condition. The attitude of these, as well as that of other health professionals, is particularly important as it may influence the way in which the person understands crucial information during the transition. They appreciated not having to retell their stories and recognized the difference in care when staff knew them (Guo et al., 2022). Telephone contact with the institution, whether through administrative support or clinical support, was a resource mentioned as a facilitator when seeking advice on what to do, which aligns with the illustrations found in the studies analyzed.

When analyzing the illustrations, different types of feelings emerge related to palliative care in the home of the person in a palliative situation: inexperience, fear, deprivation of freedom, anguish, and insecurity. The physical limitations resulting from the worsening of the disease can lead to a decrease in willingness to self-care, identified in studies as a related attitude that inhibits a successful transition.

People with low socioeconomic status have worse health indicators when compared to people with high socioeconomic status (Marmot et al., 2012). High socioeconomic status influences increased life satisfaction (Marmot et al., 2012). Meleis et al. (2000) recognize that low socioeconomic status can unfavorably inhibit the transition, conditioning access to health resources, access to information, and the ability to implement measures resulting from the new health condition.

When providing care, the lack of information regarding issues related to the worsening of the disease can lead to serious consequences, such as the worsening of the clinical condition (WHO, 2022), Some illustrations of the studies analyzed expressed this.

Palliative care intervenes in the suffering of people with serious illnesses and at the end of life through rigorous symptom control, adequate communication, support, and working side by side with the family and as a team when this family support is non-existent. Home support for patients receiving end-of-life palliative care at home depends largely on the daily presence of caregivers and their involvement in providing care, as illustrated by the participants in the studies analyzed. The growth in life expectancy and the increase in the number of older adults are consequences of rapid aging and the insufficient development of health care and state social services, resulting in gaps in the provision of end-of-life care and the burden on family caregivers (Krakowiak, 2020).

The difficulty in accessing services was a barrier highlighted by the person in a palliative situation for the continuity of home care, as illustrated in the quotes from the participants in the studies analyzed. In another study of transition experiences between care settings from the perspectives of people with advanced illness receiving specialized palliative care and their family caregivers, many participants commented on a transition's “bad” timing (e.g., emergency admissions outside working hours) and uncertainty about access to care (Guo et al., 2022).

The findings illustrate that transitions are multidimensional, involving several interconnected themes: Emotional Adaptation: Patients experience a range of emotions, from relief at returning home to anxiety about managing care responsibilities. This emotional journey reflects the internal transition they undergo as they adapt to their changing roles and health status. Logistical Challenges the transition requires significant adjustments to home environments, such as setting up medical equipment and coordinating care. These practical aspects of transition often determine the level of comfort and stability patients experience at home. Social support, including family caregivers and healthcare professionals, play a critical role in facilitating a smoother transition. Their involvement helps patients navigate the complexities of home-based care and provides a buffer against feelings of isolation. Contributions to Understanding Transitions, while the findings are descriptive, they contribute to a nuanced understanding of how transitions are experienced in home-based palliative care. Specifically: They highlight the interplay between emotional and logistical factors, showing how these elements shape patients’ overall experiences. The role of caregivers and professional support emerges as a critical facilitator of successful transitions, aligning with previous literature on the importance of integrated care models. By identifying inhibitors, such as inadequate resources or communication gaps, the findings offer actionable insights for improving transitions in palliative care settings.

The provision of home-based palliative care is shaped by diverse healthcare systems worldwide. In countries with socialized healthcare, such as many in Europe, services are often widely accessible, reducing financial barriers for patients and families. Conversely, in insurance-based systems, such as in the United States, access may depend on coverage limitations, potentially excluding some patients. These differences underscore the need to consider local contexts when applying the findings of this synthesis.

This study synthesized the lived experiences of adults receiving home-based palliative care, providing insights into the emotional, social, and logistical challenges faced during this transition. The findings highlight key facilitators, such as personalized care and strong support networks, while also identifying barriers, including limited resources and caregiver burden. These findings resonate with the principles outlined in the National Consensus Project for Quality Palliative Care (NCP) guidelines, which emphasize patient-centered care, effective symptom management, and interdisciplinary support.

This analysis shows that understanding the experiences of the person in a palliative situation at home implies a transition, a process that depends on the characterization of personal conditions, which can facilitate or impede a healthy transition. The meaning attributed is based on experiences and the perception of reality during the stay at home and determines actions, feelings, and ways of seeing and reacting to everything that is important and significant for the person in a palliative situation and their family members.

### Implications for Practice

Understanding how people in palliative situations live in their context, the needs they feel and the resources they use highlights the need for a multi- and interdisciplinary approach. However, a health professional is needed to support the management of the entire disease process, to support access and decision on which services they can or should use, thus being a figure who anticipates needs, helps to meet difficulties, and supports decisions. Establishing a training process for therapeutic self-care centered on the person, their desires, concerns, and beliefs will contribute to promoting autonomy at home and, therefore, increase the general well-being and quality of life of the person in a palliative situation. Proximity developed by the doctor and/or nurse is necessary, who have knowledge about the disease and its evolution, the management of signs and symptoms, and the context in which they reside. They can thus provide comprehensive monitoring of the person and their family, seeking to facilitate the transition process from a curative paradigm to a palliative paradigm, acting as a case manager who supports the decisions of the person and family, providing clinical knowledge, but also knowledge of the health system, seeking to contribute to the well-being of the person experiencing an incurable, advanced and progressive disease at home.

The findings underscore the need to view transitions in palliative care as dynamic and multifaceted processes. For practice, this means adopting holistic approaches that address emotional, practical, and social dimensions. For policy, it highlights the importance of resource allocation to support caregivers and enhance home care infrastructure. Future research could explore targeted interventions to address specific inhibitors identified in this review.

### Limitations

This study improved knowledge about the phenomenon. Nevertheless, the results must be interpreted with caution, and some limitations need to be recognized. For example, the fact that the studies were developed in different health systems may influence the experiences that the person in a palliative situation may express.

The thematic synthesis process integrates the interpretation of results from different researchers, making the entire process dependent on the quality of the primary analysis by the authors of the original studies.

The studies included in this thematic synthesis were conducted mainly in Anglo-Saxon and Asian cultures, and little attention has been paid to cultural, regional, and ethnic diversity with regard to the responses of the person in a palliative situation and the difference in care provision between cultures.

However, some statements reflected different health behaviors when comparing less and more developed societies.

Some results remain descriptive due to the nature of the qualitative synthesis, which is based on the original interpretations provided in the primary studies. However, this descriptive depth is necessary to capture the complexity of patient experiences during the transition to home-based palliative care.

## Conclusions

This is the first qualitative synthesis published on this specific topic, identifying the aspects mentioned by people in a palliative situation that make staying at home difficult or more manageable. The identification of these factors may be relevant for the development of person-centered health policies.

The available studies relating to people in palliative situations, developed in a home context, are usually focused on resources, accessibility to healthcare or caregiver burden, and little on the experience of people with an incurable and progressive disease. Therefore, this study intended to stand out in understanding the experience of being sick in a palliative situation at home from the patient's perspective.

The results presented here indicate that the experience of a person in a palliative situation at home is largely centered on the negative impact of the disease on their life, mainly associated with the presence and worsening of physical symptoms or the limitation in carrying out daily activities as they used to do.

Regarding perceived needs, people easily perceive physical needs, as they are easily identifiable and immediate, but they have difficulty referring to social, psychological, or spiritual needs.

Regarding strategies to deal with the disease, they were essentially focused on managing signs and symptoms, but not always the most effective.

In terms of resources to deal with the disease, people have their own knowledge and that of the people they care about; the family assumes special importance in supporting the decision and the health institutions that provide formal care.

Notably, the research allows us to understand the nature, conditions, and response patterns that characterize people's transition experience, as it constitutes fundamental knowledge for excellence in the provision of nursing care to people in transition processes.

## Supplemental Material

sj-docx-1-son-10.1177_23779608251334031 - Supplemental material for Patients’ Experiences in the Transition From Hospital to Home Palliative Care: A Systematic Review and Thematic Synthesis of Qualitative Studies Supplemental material, sj-docx-1-son-10.1177_23779608251334031 for Patients’ Experiences in the Transition From Hospital to Home Palliative Care: A Systematic Review and Thematic Synthesis of Qualitative Studies by Sara Cruz, Carla Fernandes and Bruno Magalhães in SAGE Open Nursing

sj-docx-2-son-10.1177_23779608251334031 - Supplemental material for Patients’ Experiences in the Transition From Hospital to Home Palliative Care: A Systematic Review and Thematic Synthesis of Qualitative Studies Supplemental material, sj-docx-2-son-10.1177_23779608251334031 for Patients’ Experiences in the Transition From Hospital to Home Palliative Care: A Systematic Review and Thematic Synthesis of Qualitative Studies by Sara Cruz, Carla Fernandes and Bruno Magalhães in SAGE Open Nursing
