# Supplementary material for: Patients’ Experiences in the Transition From Hospital to Home Palliative Care: A Systematic Review and Thematic Synthesis of Qualitative Studies
Source: SAGE Open Nurs. 2025 Apr 15;11:23779608251334031. doi: 10.1177/23779608251334031 (PMC12033408; doi:10.1177/23779608251334031)
Supplement: sj-docx-2-son-10.1177_23779608251334031 - Supplemental material for Patients’ Experiences in the Transition From Hospital to Home Palliative Care: A Systematic Review and Thematic Synthesis of Qualitative Studies [file sj-docx-2-son-10.1177_23779608251334031.docx]

**Supplementary data**

**Research Question / Search strategy**

**MEDLINE**

Search conducted on October 31, 2023

| **Search** | **Query** | **Records retrieved** |
| --- | --- | --- |
| #1 | ("Palliative care"[MeSH Terms] OR "Terminally ill"[MeSH Terms] OR "Terminal Care"[MeSH Terms] OR "palliative medicine"[MeSH Terms] OR "end of life care*"[Title/Abstract] OR "life care end*"[Title/Abstract] OR "Palliative Supportive Care"[Title/Abstract] OR "Palliative Therapy"[Title/Abstract] OR "palliative treatment*"[Title/Abstract] OR "palliative medicine"[Title/Abstract] OR "Palliative care"[Title/Abstract] OR "Terminal Care"[Title/Abstract] OR "Terminally ill"[Title/Abstract] OR "end-of-life care"[Title/Abstract] OR "palliative*"[Title/Abstract] OR "end of life"[Title/Abstract] OR "Terminally ill patients"[Title/Abstract] OR "Palliative treatment"[Title/Abstract]) | 160,601 |
| #2 | ("perception"[MeSH Terms] OR "attitude"[MeSH Terms] OR "perception*"[Title/Abstract] OR "attitude*"[Title/Abstract] OR "experience*"[Title/Abstract] OR "view*"[Title/Abstract]) | 3,003,772 |
| #3 | ("home nursing"[MeSH Terms] OR "residential treatment"[MeSH Terms] OR "Domiciliary care"[Title/Abstract] OR "care domiciliary"[Title/Abstract] OR "Home care"[Title/Abstract] OR "Homecare"[Title/Abstract]) | 35,583 |
| #4 | ("Palliative care"[MeSH Terms] OR "Terminally ill"[MeSH Terms] OR "Terminal Care"[MeSH Terms] OR "palliative medicine"[MeSH Terms] OR "end of life care*"[Title/Abstract] OR "life care end*"[Title/Abstract] OR "Palliative Supportive Care"[Title/Abstract] OR "Palliative Therapy"[Title/Abstract] OR "palliative treatment*"[Title/Abstract] OR "palliative medicine"[Title/Abstract] OR "Palliative care"[Title/Abstract] OR "Terminal Care"[Title/Abstract] OR "Terminally ill"[Title/Abstract] OR "end-of-life care"[Title/Abstract] OR "palliative*"[Title/Abstract] OR "end of life"[Title/Abstract] OR "Terminally ill patients"[Title/Abstract] OR "Palliative treatment"[Title/Abstract]) AND ("perception"[MeSH Terms] OR "attitude"[MeSH Terms] OR "perception*"[Title/Abstract] OR "attitude*"[Title/Abstract] OR "experience*"[Title/Abstract] OR "view*"[Title/Abstract]) AND ("home nursing"[MeSH Terms] OR "residential treatment"[MeSH Terms] OR "Domiciliary care"[Title/Abstract] OR "care domiciliary"[Title/Abstract] OR "Home care"[Title/Abstract] OR "Homecare"[Title/Abstract]) | [1,592](https://pubmed.ncbi.nlm.nih.gov/?term=%28%28%28%22Palliative+care%22%5BMeSH+Terms%5D+OR+%22Terminally+ill%22%5BMeSH+Terms%5D+OR+%22Terminal+Care%22%5BMeSH+Terms%5D+OR+%22palliative+medicine%22%5BMeSH+Terms%5D+OR+%22end+of+life+care%2A%22%5BTitle%2FAbstract%5D+OR+%22life+care+end%2A%22%5BTitle%2FAbstract%5D+OR+%22Palliative+Supportive+Care%22%5BTitle%2FAbstract%5D+OR+%22Palliative+Therapy%22%5BTitle%2FAbstract%5D+OR+%22palliative+treatment%2A%22%5BTitle%2FAbstract%5D+OR+%22palliative+medicine%22%5BTitle%2FAbstract%5D+OR+%22Palliative+care%22%5BTitle%2FAbstract%5D+OR+%22Terminal+Care%22%5BTitle%2FAbstract%5D+OR+%22Terminally+ill%22%5BTitle%2FAbstract%5D+OR+%22end-of-life+care%22%5BTitle%2FAbstract%5D+OR+%22palliative%2A%22%5BTitle%2FAbstract%5D+OR+%22end+of+life%22%5BTitle%2FAbstract%5D+OR+%22Terminally+ill+patients%22%5BTitle%2FAbstract%5D+OR+%22Palliative+treatment%22%5BTitle%2FAbstract%5D%29%29+AND+%28%28%22perception%22%5BMeSH+Terms%5D+OR+%22attitude%22%5BMeSH+Terms%5D+OR+%22perception%2A%22%5BTitle%2FAbstract%5D+OR+%22attitude%2A%22%5BTitle%2FAbstract%5D+OR+%22experience%2A%22%5BTitle%2FAbstract%5D+OR+%22view%2A%22%5BTitle%2FAbstract%5D%29%29%29+AND+%28%28%22home+nursing%22%5BMeSH+Terms%5D+OR+%22residential+treatment%22%5BMeSH+Terms%5D+OR+%22Domiciliary+care%22%5BTitle%2FAbstract%5D+OR+%22care+domiciliary%22%5BTitle%2FAbstract%5D+OR+%22Home+care%22%5BTitle%2FAbstract%5D+OR+%22Homecare%22%5BTitle%2FAbstract%5D%29%29&ac=no&sort=relevance) |
| #5 | ("Palliative care"[MeSH Terms] OR "Terminally ill"[MeSH Terms] OR "Terminal Care"[MeSH Terms] OR "palliative medicine"[MeSH Terms] OR "end of life care*"[Title/Abstract] OR "life care end*"[Title/Abstract] OR "Palliative Supportive Care"[Title/Abstract] OR "Palliative Therapy"[Title/Abstract] OR "palliative treatment*"[Title/Abstract] OR "palliative medicine"[Title/Abstract] OR "Palliative care"[Title/Abstract] OR "Terminal Care"[Title/Abstract] OR "Terminally ill"[Title/Abstract] OR "end-of-life care"[Title/Abstract] OR "palliative*"[Title/Abstract] OR "end of life"[Title/Abstract] OR "Terminally ill patients"[Title/Abstract] OR "Palliative treatment"[Title/Abstract]) AND ("perception"[MeSH Terms] OR "attitude"[MeSH Terms] OR "perception*"[Title/Abstract] OR "attitude*"[Title/Abstract] OR "experience*"[Title/Abstract] OR "view*"[Title/Abstract]) AND ("home nursing"[MeSH Terms] OR "residential treatment"[MeSH Terms] OR "Domiciliary care"[Title/Abstract] OR "care domiciliary"[Title/Abstract] OR "Home care"[Title/Abstract] OR "Homecare"[Title/Abstract]) AND ("qualitative research"[MeSH Terms]) | **223** |

**CINAHL (EBSCO)**

Search conducted on October 31, 2023

| **Search** | **Query** | **Records retrieved** |
| --- | --- | --- |
| #1 | AB (MH "Palliative Medicine" OR MH "Palliative Care" OR MH "Terminally ill" OR MH "Terminal Care" OR "end of life care*" OR "life care end*" OR "Palliative Supportive Care" OR "Palliative Therapy" OR "palliative treatment*" OR "palliative medicine" OR "Palliative care" OR "Terminal Care" OR "Terminally ill" OR "end-of-life care" OR "palliative*" OR "end of life" OR "Terminally ill patients" OR "Palliative treatment") | 75,567 |
| #2 | AB (MH "experience" OR MH "Perception" OR MH "Attitude" OR "perception*" OR "attitude*" OR "experience*" OR "view*") | 737,559 |
| #3 | AB (MH “Domiciliary Care” OR MH "Home Nursing" OR "Domiciliary care" OR "care domiciliary" OR "Home care" OR "Homecare") | 16,660 |
| #4 | #1 AND #2 AND #3 | 668 |
| #5 | #1 AND #2 AND #3 AND (MH "Qualitative Studies") | **186** |

**PsycINFO (EBSCO)**

Search conducted on October 31, 2023

| **Search** | **Query** | **Records retrieved** |
| --- | --- | --- |
| #1 | AB (DE "Palliative Care" OR DE "Terminally Ill Patients" OR "Palliative Medicine" OR "Terminal Care" OR "end of life care*" OR "life care end*" OR "Palliative Supportive Care" OR "Palliative Therapy" OR "palliative treatment*" OR "palliative medicine" OR "Palliative care" OR "Terminal Care" OR "Terminally ill" OR "end-of-life care" OR "palliative*" OR "end of life" OR "Terminally ill patients" OR "Palliative treatment") | 26,207 |
| #2 | AB (DE "Perception" OR DE "Attitudes OR "Attitude*" OR "perception*" OR "experience*" OR "view*") | 36,924 |
| #3 | AB (DE "Home Care" OR "Home Nursing" OR "Domiciliary care" OR "care domiciliary" OR "Home care" OR "Homecare") | 11,590 |
| #4 | #1 AND #2 AND #3 | **3** |

**ProQuest – Dissertations and Theses**

Search conducted on October 31, 2023.

("palliative care") AND ("Home nursing") AND ("Perception")

Filters: Dissertations & Theses

**Records retrieved**: 28

**Worldcat**

Search conducted on October 31, 2023.

kw:"palliative care" AND kw:"perception" AND kw:"home nursing"

Filters: Tese, Dissertação

**Records retrieved**: 1

**Joanna Briggs Institute Appraisal Tool and Scoring System**

| **Studies / citations** | **Q1** | **Q2** | **Q3** | **Q4** | **Q5** | **Q6** | **Q7** | **Q8** | **Q9** | **Q10** | **Score** |
| --- | --- | --- | --- | --- | --- | --- | --- | --- | --- | --- | --- |
| (Appelin and Berterö, 2004) | Y | Y | Y | Y | Y | N | Y | Y | Y | Y | 9 |
| (Ushikubo, 2005) | Y | Y | Y | Y | Y | N | N | U | Y | Y | 7,5 |
| (Artsanthia et al., 2011) | Y | Y | Y | Y | Y | N | N | U | Y | Y | 7 |
| (Wallin et al., 2015) | Y | Y | Y | Y | Y | N | Y | Y | Y | Y | 9 |
| (Hirakawa et al., 2017) | Y | Y | Y | Y | Y | N | Y | Y | Y | Y | 9 |
| (Rocío et al., 2017) | Y | Y | Y | Y | Y | N | Y | Y | Y | Y | 9 |
| (Aebischer Perone et al., 2018) | Y | Y | Y | Y | Y | N | U | Y | Y | Y | 8 |
| (WennmanRingheim and Wijk, 2020) | Y | Y | Y | Y | Y | N | Y | Y | Y | Y | 9 |
| (Dillen et al., 2021) | Y | Y | Y | Y | Y | N | Y | U | Y | Y | 8 |
| (Fu et al., 2021) | Y | Y | Y | Y | Y | N | N | Y | Y | Y | 8 |
| (Isenberg et al., 2021) | Y | Y | Y | Y | Y | N | Y | Y | Y | Y | 9 |
| (Liu et al., 2021) | Y | Y | Y | Y | Y | N | Y | Y | Y | Y | 9 |
| (SalifuAlmack and Caswell, 2021) | Y | Y | Y | Y | Y | N | Y | Y | Y | Y | 9 |
| (Prado et al., 2022) | Y | Y | Y | Y | Y | N | Y | Y | Y | Y | 9 |
| (Ahmed et al., 2023) | Y | Y | Y | Y | Y | N | N | Y | Y | Y | 8 |
| (BroseWillis and Morgan, 2023) | Y | Y | Y | Y | Y | N | N | Y | Y | Y | 8 |
| (Pérez Sandoval et al., 2023) | Y | Y | Y | Y | Y | N | N | Y | Y | Y | 8 |
| % | 100% | 100% | 100% | 100% | 100% | 0% | 59% | 88% | 100% | 100% |  |

**Key:** Y=Yes, N=No, U=Unclear, Qn – number of Question

**Questions:**

Q1. Is there congruity between the stated philosophical perspective and the research methodology?

Q2. Is there congruity between the research methodology and the research question or objectives?

Q3. Is there congruity between the research methodology and the methods used to collect data?

Q4. Is there congruity between the research methodology and the representation and analysis of the data?

Q5. Is there congruity between the research methodology and the interpretation of the results?

Q6. Is there a statement locating the researcher culturally or theoretically?

Q7. Is the influence of the researcher on the research, and vice-versa, addressed?

Q8. Are participants, and their voices, adequately represented?

Q9. Is the research ethical, according to current criteria, or for recent studies, and is there evidence of ethical approval by an appropriate body?

Q10. Do the conclusions drawn in the research report flow from the analysis, or interpretation, of the data?

**Joanna Briggs Institute ConQual Score**

Determination of the study’s credibility/dependability within the findings

(Munn, Porritt, Lockwood, Aromataris, & Pearson, 2014)

| **Systematic review title** | The experiences of adult palliative patients in home palliative care: a qualitative systematic review of the literature. | | | | | |
| --- | --- | --- | --- | --- | --- | --- |
| **Population** | Adult (≥ 18 years) home care palliative patient. | | | | | |
| **Phenomena** | Experience and perceptions of adult home care palliative patient | | | | | |
| **Context** | Adult (≥ 18 years) home care palliative patient  Studies in which participants were solely and exclusively exposed to home care | | | | | |
|  | **Synthesised finding** | Type of research | Dependability | Credibility | ConQual Score | Comments |
| (Appelin & Berterö, 2004) | **Safe but Unsafe at Home**  *... It’s easier to be unwell if I can stay at home ...*  *we embrace each other frequently and a great deal and it feels safe and secure ...*  *... we are able to be together and it makes us happy in the midst of the sorrow ...*  **A Sense of Powerlessness**  *I have asked once in a while if they can do anything ... but they did not answer me really ...*  **Change of Everyday Life**  *... I want to live as usual but it’s not possible ... I can’t cook ... I can’t go for a walk ...”*  *it’s not a good feeling that she has got both our jobs now*  *...there are a lot of things which could be planed more calmly, but ...*  *...when you are healthy it is ... not your way of thinking I’m afraid ... you take everything for granted.*  **Hope and Belief in the Future**  *... but I shall go on fighting ... hm ... I have a feeling that my life is not finished yet ...*  *I think that ... It can be painful and hard with the pain ...but I hope I do not have to be alone at the time...*  *I am aware that it doesn’t cure but I hope it can bring relief ... so it’s a hopeful feeling anyway.*  **Essential Meaning—Uncertain Safety**  *... well I ... it feels pleasant and I feel quite well here, so I am...satisfied...but I don’t want to be a burden if I get worse...*  ...if I get worse I have to go to the hospital...I can’t stay at home then...it is so... | Qualitative - High | Unchanged | Remains  unchanged | High | High: the dependability score is based on the specific questions from the critical appraisal scores for included studies related to the appropriateness of the conduct of the research with research aims and purpose 4-5 ‘yes’ responses, the paper remains unchanged  Remains unchanged as all findings  unequivocal |
| (Ushikubo, 2005) | **Symptoms management**  In the field of physical symptoms and daily life disorders, it has become clear that patients are suffering from symptoms such as ``sickness'' and ``pain of not being able to take care of oneself.  **Gratefulness toward being able to perform some Physical functions**  Although he was suffering from severe illness, he was supported by the fact that he was able to control his symptoms and was grateful for his preserved physical functions.  **Renewed spirits in facing up to their illness**  In addition, ``motivation to fight illness,'' ``faith,'' ``hardworking personality,'' and ``hope'' have all been associated with treatment from the early stage of diagnosis to the present. This has been shown to be a fundamental element of support for caregivers.  **Achieving their roles within their families**  Furthermore, in role performance, although some people suffer from the feeling of not being able to fulfill their assigned role, they are able to do what is possible, such as fulfilling their role within the family. They were performing different roles, which became a source of competition in their lives.  **Hope**  Although they feel like they have given up, they also have hope, such as the hope that things will get better, and the hope that new drugs will be developed. It became clear that he was living a life of.  **Sense of relief derived from medical services**  My gratitude for the support I received regarding my physical symptoms and life disorders, gratitude for the love of my family, the sense of security that came from the home medical care system, and the kindness of the people around me.  **Gratitude for the thoughtfulness of their families**  On the other hand, he felt sorry for his family, but he also expressed gratitude for the love he received from his family, including gratitude for taking care of him.  **Gratitude for the consideration of others**  While talking about their feelings, they also felt gratitude for the kindness and support of those around them, such as ``I am grateful for the casual consideration of my neighbors,'' and this became a source of support for their lives.  **Religious beliefs**  In particular, ``anxiety due to being close to death'' was frequently heard from MS patients who had experienced emergency medical treatment several times due to deterioration of their condition. Anxiety about an uncertain future, combined with other afflictions, increased the severity of each affliction.  **Unconquerable personality**  As mentioned above, the perception of the disease changed as the treatment progressed, but in the early stages of diagnosis, patients reported thoughts of suicide and feelings of despair if something happened to them. At the same time, I felt that I wanted to get better somehow. | Qualitative - High | Downgrade 1 level  – Moderate | Downgrade 1 level | Moderate | Downgraded one level as  scored 2–3 out of 5 for questions relating to appropriateness of conduct of the research.  Downgrade 1 level: the synthesized finding contains a mixture of unequivocal and equivocal findings. |
| (Artsanthia, Mawn, Chaiphibalsarisdi, Nityasuddhi, & Triamchaisri, 2011) | **Tremendous Suffering**  During the in-depth interviews, the end-stage renal disease (ESRD) participants shared many problems as their symptoms progressed over time. They shared stories of tremendous suffering including severe bone pain due to poor circulation, which led to gangrene and amputation of one man’s penis.  **Economic Consequences**  As one participant noted, it was quite difficult to become accustomed to this disease that causes physical suffering and resulted in significant economic consequences. Many were forced to retire early.  **Inadequate Community Support**  Another major finding from the interviews with ESRD participants and their family members was that supportive services in the community were not adequate. In addition, several noted that teamwork among clinic staff and home- based care services in the community was not effective, which also resulted in a diminished capacity to care for their loved ones.  **Concern for the Future**  For many families, the diagnosis of a life-threatening illness of a family member was their first major confrontation with death. | Qualitative - High | Downgrade 1 level  – Moderate | Downgrade 1 level | Moderate | Downgraded one level as  scored 2–3 out of 5 for questions relating to appropriateness of conduct of the research.  Downgrade 1 level: the synthesized finding contains a mixture of unequivocal and equivocal findings. |
| (Wallin, Carlander, Sandman, & Hakanson, 2015) | **Resisting Death by Eating**  Ruth described how she experienced changes in taste and decreased appetite and was discomforted by smell. She was fed up with the sip feed, so she decided to eat ordinary food instead, even though it “tasted like paper,” because she realized she needed the calories: “*It won’t do otherwise - you have to eat to survive (. . .) so I just had to decide to do it.”*  Cathleen, in an advanced stage of gynecological cancer and suffering from decreased appetite, taste alterations, and nausea.  Well, it is, of course, a feeling of kind of (. . .) *I’m go- ing to die of this, you know. The cancer just carries on eating up my body, but so far, there are quite a lot of parts that haven’t been filled with cancer. The more I lose weight, the less there is of me that is healthy. So, there’s kind of a resistance (. . .) being able to kind of keep (. . .) hold of [life].*  **Struggling with Social Gatherings Around Food**  For Paul, suffering from ALS, immobilization, and dysphagia illustrated this. He and his wife used to share meals, but his wife now had to feed him with enteral nutrition through a feeding tube at the same time as trying to finish her own meals:  *It’s a matter of deciding then—is she going to eat while her food is hot, will we do it afterwards, at the same time and so on. It depends a little on what we want. (. . .) Yes, and there’s never really a feeling of togetherness in that—there just isn’t.*  *We had guests for lunch here, and, I suppose, I was keeping up okay with the soup, but then when we got to the main course, I wasn’t hungry anymore. (. . .) And the company wasn’t any good either it looks like I don’t think the food is any good.*  **Eating to Please and Unburden Others**  This was illustrated by Cathleen, who had decreased appetite and stomach pain due to gynecological cancer. She talked about her experiences of having her anxious parents visiting and bringing sweets: *“Yes, of course, it makes you feel obliged to eat those things you don’t want to eat, to please other people and make them happy.”*  Edward, for example, who could only eat very small amounts of food because of all the troubles related to his pancreas, expressed the following:  *She’s taken over [responsibility for food] completely. I’m really grateful for that; otherwise, eating would have been completely disorganized. If I had lived alone, I would have been dead by now. I’m pretty sure of that.* | Qualitative - High | Downgrade 1 level  – Moderate | Downgrade 1 level | Moderate | Downgraded one level as  scored 2–3 out of 5 for questions relating to appropriateness of conduct of the research.  Downgrade 1 level: the synthesized finding contains a mixture of unequivocal and equivocal findings. |
| (Hirakawa, Chiang, Hilawe, & Aoyama, 2017) | **Anxiety about the future**  *I want to remain at home as long as possible and avoid being hospitalized. (Sixty-seven, female, others)*  *I really dislike hospitals. (Eighty-three, male, others)*  *Loneliness is unavoidable as we grow older.* *(Seventy-eight, female, one-person household)*  *I cannot ask my son for care support because I do not feel at ease with my daughter-in-law.* *(Eighty-seven, female, others)*  If I develop severe dementia, I would like to be institutionalized to avoid becoming a burden on my wife. *(Seventy-two, male, others)*  *I worry that I may not be able to discuss care services with the hospital or care service center if my condition deteriorates. (Seventy-five, male, one-person household)*  **Abandonment of control**  *I think I would have no choice but to be institutionalized in the future, just as my sister was. (Eighty-seven, male, others)*  *I do not know what I am going to do until I am in that situation. (Seventy-five, female, one-person household)*  *I do not want to think about it for now. (Seventy-five, female, one-person household)*  **Precarious mutual support**  *I want to stay with my wife and care for her even though she has dementia. (Eighty-one, male, older couple only household)*  *I have to go on with daily life and continue caring for my wife despite my worries about her future. (Seventy, female, older couple only household)*  **Delegating decision-making**  *Because I live alone and have financial difficulties, I want to leave a power of attorney to my children. (Seventy-eight, female, one person household)*  *I have never thought about it, but I hope my brother will take care of me in the last days of my life. (Seventy-four male, one-person household) (Seventy-four male, one person household)*  **Clinging to current daily life**  *I am grateful that my children, daughter-in-law, and day service center staff kindly take turns caring for me.*  *I want to stay in the house where my husband and I built a life together despite the fact that he was rigid. (Eighty-seven, female, others)*  *I would be happy if my son and his family came back to live with me in the future. (Eighty-three, female, one-person household)*  *I want to stay in the house where my husband and I built a life together despite the fact that he was rigid. (Eighty-eight, female, others)*  *I want to stay in the house that I built until the very end of my life. (Eighty-seven, male, others)* | Qualitative - High | Unchanged | Remains  unchanged | High | High: the dependability score is based on the specific questions from the critical appraisal scores for included studies related to the appropriateness of the conduct of the research with research aims and purpose 4-5 ‘yes’ responses, the paper remains unchanged  Remains unchanged as all findings  unequivocal |
| (Rocío et al., 2017) | **Life changes**  *‘It is hard work, especially the way we have to do it’.* *C1E1*  **Caring actions**  *´When he had the catheter, I would administer the medicine, but a few times he would do it, I help him with the curing procedures, all that, help him to vacate the colostomy and those things I have already learnt how to manage´.*  *C4E1*  **Emotional and physical burden**  *‘I am filled with angst, when he says this or that ...’* *C3E3*  *‘That makes more at ease and, well, I know that with the few people that I can count on in my family, we support each other, so I know he will be well, that is what is important’.*  *C1E1*  **Physical discomfort and pain**  *‘The pain seems worse, yes, because it often does not stop – not even at night. Perhaps only once it would cease but the rest of the time it would not, at dawn the pain would calm down for some time and he was well without pain, but it would return again’.*  *C4E4*  *‘Lots of pain, especially in the lower limbs”.*  *C4E4*  **Coping**  *‘For her it has been difficult, difficult to wear a nappy and it is even more difficult for me; she is my mother’.*  *C3E1*  *‘I don’t understand why didn’t they do the chemo, why didn’t they do chemo, that is what I wonder every day, why? Why?’*  *C3E1*  **Bond between the individual in palliative care and their caregiver (dyad)**  *‘My mother sometimes thinks I scold her because I tell her to be patient, but I tell her: “mom let’s not get too anxious”’.*  *C2E2*  *‘I think my mother wants to die at home’.*  *C3E3*  **Expected responses**  *‘I’d say they should offer him support, to indulge him more, to help me, and keep us company’.*  *C4E4*  *‘I still need to know how to manage those things, I have to pay attention. Although he also pays attention and he knows how his things are applied, the colostomy and all also, he has learnt some things’.*  *C5E5*  *‘The oxygen stops a lot, they ordered medications, we were discharged and at this time we have not gotten authorizations for any of them, not even morphine, nothing’.*  *C1E1* | Qualitative - High | Unchanged | Remains  unchanged | High | High: the dependability score is based on the specific questions from the critical appraisal scores for included studies related to the appropriateness of the conduct of the research with research aims and purpose 4-5 ‘yes’ responses, the paper remains unchanged  Remains unchanged as all findings  unequivocal |
| (Aebischer Perone et al., 2018) | **Lack of autonomy**  *«I depend on my family, especially my wife. I can just turn myself in the bed. My wife feeds me, I cannot get up, I lose my mind, I often forget things and have difficulties to speak. » (P6Q3a, M, 60–69, urban setting, lives with his relative)*  *«I cannot move, pain exhausts me. I have no strength. I did not get help. They do not know how to help me. » (P11Q5a, M, 70–79, urban setting, lives with his relative)*  **Loneliness, depression, and social exclusion**  *« We are both not well. I am exhausted and we both do not enjoy our lives any more. I wish we both die together and I wish it happens soon. » (P25Q4a, F, 50–59, urban setting, lives with her relative)*  *« I cry, I lay down all the time in bed. This is no life anymore. I do not enjoy my life at all. My husband as well. I wait for death and that is it. » (P28Q4a, F, 80–89, rural setting, lives with her relative)*  *« We are isolated, people avoid us. Can people with this problem meet, or have volunteers to visit us and make us feel like human? » (P2Q4d, F, 80–89, urban setting, lives alone)*  *« People know I am seriously ill and they either avoid me or feel sorry about me. I am not happy about it. It makes me forced to isolate myself from people. I am better off alone. » (P58Q2a, M, 60–69, rural setting, lives with his relative)*  *« I believe in God and he helps me when I feel the worst. I have regular visits from the priest. » (P24Q12a, M, 30–39, urban setting, lives with his relative)*  *« I did not solve those issues yet. Maybe I am angry at God because of all that has happened to me. I did not look for help. It is something I have to deal with myself. » (P18Q12a, M, 80–89, urban setting, lives with his relative)*  **Dissatisfaction with pain control and symptoms management**  *« My pain is strong and I would like sometimes to jump out of the window. Patients with such pain should have proper therapy. » (P1Q7c, F, 70–79, urban setting, lives with her relative)*  *« I have strong pain. I buy pain killers. My doctor prescribes medication, but they help only for a short while. I become immune to this medication. I buy some pain killers that help my friends too. » (P16Q7a, F, 70–79, urban, lives with her relative)*  *« I did not ask for help. My disease influences my breathing. These breathing problems cause many difficulties. I know this is the consequence of my diagnosis. » (P13Q6a, F, 40–49, urban, lives with her relative)*  *« I need support for my wounds, not for the diarrhoea. I need advice about what to do. I suffer because of diarrhoea’s consequences. » (P27Q10b, M, 50–59, urban setting, lives with his relative)*  **Burden on families**  *« My family worries more about me. I also worry about them. I am concerned on how they cope now and what will happen with them after I die. » (P27Q15a, M, 50–59, urban setting, lives with his relative)*  *«I worry about my mum, she is exhausted. It is difficult to care for me. I worry about the financial situation of my family. I did not get sufficient support, just basic. » (P9Q15a, M, 40–49, rural setting, lives with his relative)*  *« He could not even get a free wheelchair. He is neglected by the society. » (P14Q2a, M, 20–29, urban setting, lives with his relative)*  *« Our families need better support, especially those who are all the time with us. My husband is exhausted. My daughter studies in Banja Luka and she comes from time to time. My husband takes care of everything. He works and earns for us. » (P13Q3b, F, 40–49, urban setting, lives with her relative)*  **Deontology, professional approach, and dignity of patients**  *« I do not want to go to the hospital. I feel bad there. They treat me bad; they just give me medication, and nobody cares about me there. » (P18Q1c, M, 80–89, urban setting, lives with his relative)*  *« Help patients have dignity in the last days of their life. We need better home care. » (P3Q2c, M, 80–89, urban setting, lives with his relative)*  *« I am satisfied. The nurse comes once a month, she brings the doctor sometimes. The nurse is our blessing, we would be lost without her. » (P55Q1d, M, 50–59, rural setting, lives with his relative)*  *« Thank God I had support from my daughter. I think that support only comes from the family. I feel bad about the patients who do not have family or lack their support. Every patient should have human support and understanding. » (P35Q2b, F, 70–79, urban setting, lives alone)*  **Lack of information**  *« I have some problems with my brain. I do not know what the problem is. Something appeared on my CT. » (P26Q1a, M,60–69, urban setting, lives with his relative)*  *« We are all lost. He is not talking or moving. He is completely paralyzed. Nobody told us what to do. He was sent home to die. We ask ourselves what to do now. » (P10Q1e, M, 50–59, rural setting, lives with his relative)*  *« It is difficult to have this disease. It is even more difficult to go through all procedures. This kind of patients are ill and still they have to organize everything themselves. » (P35Q1e, F, 70–79, urban setting, lives alone)*  *« There should be a counselling service for cancer patients. Many people have cancer, it is like an epidemic. » (P36Q1f, M, 40–49, urban, lives with his relative)* | Qualitative - High | Downgrade 1 level  – Moderate | Remains  unchanged | Moderate | Downgraded one level as  scored 2–3 out of 5 for questions relating to appropriateness of conduct of the research.  Remains unchanged as all findings  unequivocal |
| (Wennman, Ringheim, & Wijk, 2020) | **Create a Safe Environment**  *They can’t see the notes . . . it’s probably wrong, because they can’t see the patient records . . . unable to solve it with any consultant— or log in, so it’s a pure stone-aged action to sit and send a test result and other stuff by fax. So that’s too bad.*  **See the Person**  *And I actually discussed the matter with the doctor here, we sat down and talked about it for almost an hour, about how things would turn out and how it (dying) would be. She said, “We are here for you all the way.” “All the way,” now I know exactly what they mean.*  **Better to Manage Care at Home**  I *only get reminded of a lot of negative things by visiting the hospital. For me it’s just, if I go to the hospital, I have to recover during the following 24 hours, it is nothing but negative...it’s much better if everything could be managed here (at home).* | Qualitative - High | Unchanged | Remains  unchanged | High | High: the dependability score is based on the specific questions from the critical appraisal scores for included studies related to the appropriateness of the conduct of the research with research aims and purpose 4-5 ‘yes’ responses, the paper remains unchanged  Remains unchanged as all findings  unequivocal |
| (Dillen et al., 2021) | **Availability**  *SPHC (specialist palliative home care) patient: The accessibility of the SPHC team is a psychological relief, a great feeling knowing that you can reach someone at all times. So far, I have not yet needed it. I see it as an emergency plan.*  *SPHC patient: They told us we will come over immediately. And indeed, they were here in half an hour, regardless of the time of day or night. And that is the advantage, that people do not die of fear.*  **Provision of information/education**  *GPHC (generalist palliative home care) patient: That they do not provide me with false reassurances, but that they disclose everything they know, everything they know but the patient does not know, by doing so the patient receives a gift... the consultation was honest and great.*  **Professional competence**  *SPHC patient: ... you get the feeling that they dedicate plenty of time, much more experience in the, let’s say terminal stage of cancer, uhm, this by itself conveys a great sense of security. You always have the time to ask whatever is on your mind and you get the feeling the people do not leave (laughs) until it is all sorted out and that, uhm, makes it a lot easier. By doing so, pain management worked out great for me.*  **Patient empowerment**  *SPHC patient: Yes, I have learned a lot here, to say, I am writing a doctoral thesis (laughs)... the doctor even told me, the nurse would not have been able to do this.*  **(Person) of trust**  *SPHC patient: And I believe that this is something incredibly important, that they are actually listening, searching for solutions, searching for solutions with me, so not somehow against me, but, I think they have figured out my personality rather quick (laughs). And that was, well, that made it easy for me to gain trust very quickly.*  **Comprehensive responsibility**  *GPHC family caregiver: We are under a lot of stress, so once in a while you use curse words, uhm, my mother got sick as well, so my mother stayed in one room, my father in... to be honest, that was nerve-wracking. But the palliative home care team has really helped us out a lot with that. They told us, whenever you need our help, just let us know, we are here for you and they actually comforted my mother as well. It is the small gestures that count.*  **External collaboration**  *SPHC patient: They have taken this over themselves, the SPHC team called and told them the patient is in our care now, that they do not have to come anymore for the time being, that they are on hold, on a waiting list, on whatever, standby for now, in case they would be needed again, but that for now the SPHC team is taking over. That was excellent because I did not have to take care of anything.*  **Internal cooperation**  *GPHC patient: Well, security and, uhm, that they work with each other, learn from each other’s experience and share their knowledge. This gives me the reassurance that I am in good hands.*  **Direct communication**  *SPHC family caregiver: I can always send Dr. <name > a whatsapp or SMS. For example, in August my mom developed skin metastases and once 10. the first one was visible, I sent her a picture. She replied, no, she does not believe this is an abscess but a metastasis. And she would like to come over and take a look... so really, just the communication, the security of having a contact person* | Qualitative - High | Downgrade 1 level  – Moderate | Remains  unchanged | Moderate | Downgraded one level as  scored 2–3 out of 5 for questions relating to appropriateness of conduct of the research.  Remains unchanged as all findings  unequivocal |
| (Fu et al., 2021) | **Management of Exacerbations**  *It was her stats; her oxygen levels were all over the place. Every time she went in it was 999 [emergency call] and one occasion it was the helicopter. It was a rush job.* [H.C02, carer]  **Needs for Palliative Care**  *Often the best palliative care is not provided because there isn’t that step back and that holistic assessment in a timely fashion.* [L.HP09, hospital consultant]  *Often the best palliative care is not provided because there isn’t that step back and that holistic assessment in a timely fashion. [H.HP08, hospice consultant]*  **Access and Pathways**  *I’ve got my insurance sorted so the family don’t pay for the funeral, but I would, definitely want to be in a hospice, if it was possible. [L.P04, patient]*  *From a specialist service, [the questions are] what are we actually offering and who is best to manage those acute exacerbations? [L.HP05, hospice consultant]*  **Integration of Palliative Care**  The combined integrated working with the palliative MDT has helped enormously . . . we’re more aware of each other’s roles and how that works in together. [L.HP11, respiratory nurse specialist]  Other areas use the structure as an excuse not to do that. It’s like it’s a barrier of reasons why not to . . . it’s a kind of ‘can’t do’ culture [H.HP01, respiratory advanced nurse practitioner]  I think one of the worst hurdles for us is staffing, the gaps in posts and recruitment. [H.SM01, district nurse lead] | Qualitative - High | Downgrade 1 level  – Moderate | Remains  unchanged | Moderate | Downgraded one level as  scored 2–3 out of 5 for questions relating to appropriateness of conduct of the research.  Remains unchanged as all findings  unequivocal |
| (Isenberg et al., 2021) | **Health and Well-Being**  *Some patients’ health was worse than anticipated once they arrived home; one expressed he did not expect to “experience so much fatigue” (Patient 62M).*  *Several participants expressed feeling secure in the hospital because of access to continuous nursing care and therefore felt “apprehensive” (Patient 48F) about going home: “I’m scared because I’m not going to have somebody [in the home] 24/7. . . what if I fall, if somebody’s not there. I’d get hurt” (Patient 51F).*  *“After a couple days [I realized] this ain’t so bad****. I*** *can manage here by myself.”* *(Patient 51F).*  **Practical Needs**  ***Transportation***  *Well, a lot of those ambulance people, they are quite something. Quite organized and better than what they used to be because you’re not doing well if you don’t know when if you’re coming back home you’ve got the voyager chairs . . .stretcher as well as a chair if you’re able to sit. And I thought that was quite interesting because I’ve never been in one before that. I’ll say where’d you get that chair, I can use that at home. (Patient 82F)*  ***Setting Up the Home for Care***  *I thought we would be okay, but it’s only when he got home, and he had to lie on this chesterfield which is narrow. Then I recognized that things are different and that I need to rearrange everything in the room, get more equipment. I wish I had known that before. (Caregiver 82F)*  ***Healthcare Providers in the Home***  *It won’t be care in the sense of hands on care. He [physician] won’t be changing diapers or linen... I think he’ll be monitoring the effects of his medication, his vital signs and he’ll probably have a baseline of his cognitive ability. So, he’ll know if it’s going down. I hope the physician comes at least once but is available if I need to talk. (Caregiver 82F)*  *“If there are any concerns, we just put a call through and. . . he’ll either provide a solution over the phone or he’ll say I’ll be out in 15 minutes” (Caregiver 74M).*  *I know any nursing care I need, if I need that drain- age tube drained, they’ll come in and do that, they’ll change bandages. . . I know helping with dressing, helping with baths if I need it.* (Patient 70F)  **Enablers and Disablers**  ***Caregiver’s Role***  *No, he [husband] handles....Without him I’d be dead. (Patient 77F)*  *“I’ve had to do the scheduling. I’ve had to deal with the [personal support worker], deal with the nurs- ing, deal with what he needs for equipment” (Caregiver 75F).*  ***Community Support***  *“was happy to be with them and have a chat with them. . . we get those emotional support from our parish commu- nity” (Caregiver 69F).*  ***Education***  *I had to have a plan. . .because they were coming in and out of the hospital room 13 times [a day]. . . so the pharmacist came and he gave me a plan of when to give drugs and how much. . . once I got home, the nurse helped me scale it down to like six times, and the doctor helped too... I can’t imagine having to do 13 or 14 different things in a day. (Caregiver 64F)*  *No. . .Because I can't evaluate his condi- tion. So if his condition were to deteriorate and really required a hospitalization, I'm not in a posi- tion to make that call. (Caregiver 63F)*  ***Communication and Coordination***  *It was disorganized. There was some mis- communication. . . I ended up staying in the hospital the entire weekend unnecessarily because one of the doctors who didn’t really know me very well, said “Yeah, someone’s going to come in to see you week- end.” Well no one ever did. So they just let me go on Monday. (Patient 59M)*  *I don’t know who I was talking to, I hear one name, I hear another... And then I’d have voices on the phone and I’m, like who’s this, which one is that? Holy smokes. Come on, just stick to one or two peo- ple... because they’re all asking the same questions. (Caregiver 58M)*  *This lack of coordination led one caregiver to describe the overall transition process as “rudderless” (Caregiver 57M).*  ***Uncertainty***  *“I didn’t know anything and I wasn’t being taught any- thing on how to handle my own condition. You know, so it’s a little scary” (Patient 77F).*  *Things are always clear until you have to do it your- self. If she’s in pain...what do you do? And how quickly are you allowed to give the breakthrough medication? And are there limits to this if she’s feel- ing more pain than before? (Caregiver 48M)*  ***Financial Resources***  *I think the commode chair, we had it for 28 days. That will be ending this week, and they want a ridic- ulous price, like $50 a month to rent. . .we’re going to have to figure out what we’re going to do. (Caregiver 74M)*  *I have to budget too. . .Because every week I get somebody for Saturday and Sunday. . .So I allocated four weekends so I need at least $400 and then the rest I will allocate it for the diapers and all that she needs. . .It’s a lot to manage. (Caregiver 69F)* | Qualitative - High | Unchanged | Remains  unchanged | High | High: the dependability score is based on the specific questions from the critical appraisal scores for included studies related to the appropriateness of the conduct of the research with research aims and purpose 4-5 ‘yes’ responses, the paper remains unchanged  Remains unchanged as all findings  unequivocal |
| (Liu et al., 2021) | **Physical need**  *I feel so much pain every day. I couldn’t have a rest for a long time because of the pain (Participant 12). After the activity, I often feel very painful, but I try my best to endure pain due to fear of drug addiction (Participant 14).*  *My family unanimously decided to insist on antitumor therapy. I wish to alleviate the pain and prolong life as long as possible (Participant 10).*  **Psychological experience**  *I often forgot and didn’t realize I was a patient. When my peer was upset and sad, I would enlighten them and make them happy and relaxed (Participant 5).*  *When I was diagnosed with lung cancer, I couldn’t ac- cept it at that time. Now my mood is relatively calm, because I have no choice but to accept it (Participant 11). There is no use crying over the illness. I pretend to be optimistic with my family. Actually, I am deeply anx- ious and sad every day (Participant 12).*  **Spiritual need**  *I still do light housework, such as washing dishes and sweeping floors. Here, I think everything is still as usual.... (Participant 1).*  *When I got along with my friends, I did not mention my illness to “save face”, and they pretended not to know I was sick. It’s good for us (Participant 5).*  *I am very worried about my wife’s life after my death. She is a sensitive and fragile woman (Participant 1).*  *I want to alleviate the pain and prolong my life. I seldom think of the prognosis or death (Participant 6).*  *I suffer extreme pain every minute. I want to end my pain with euthanasia (Participant 15).*  **Social support**  *Once I attended a classmate’s wedding, and some peo- ple were afraid to have dinner with me due to fears of cancer contagion (Participant 13).*  *When I passed them (my friends), they pretended not to see me. They worried I would ask them for money. Actually, I didn’t (Participant 11).*  *Over the past three years, I had received more than 20 chemotherapy treatments. The expensive medical bur- den forced us to sell our only house and borrow money from our relatives and friends, racking up medical bills of more than 1 million RMB (Participant 6).*  *I lived in a rural area, and the rural new cooperative medical scheme could reimburse only 30%. The reim- bursement rate was too low to maintain anticancer ther- apy (Participant 9).*  **Informational need**  *After six months of treatment, the condition was not getting better. I begged and urged my wife to tell me the truth (Participant 7).*  *My wife replaced a colostomy bag for me yesterday. I found a macerated peristomal site with surrounding ex- crement. We had to go back to the hospital (Participant 15).* | Qualitative - High | Unchanged | Remains  unchanged | High | High: the dependability score is based on the specific questions from the critical appraisal scores for included studies related to the appropriateness of the conduct of the research with research aims and purpose 4-5 ‘yes’ responses, the paper remains unchanged  Remains unchanged as all findings  unequivocal |
| (Salifu, Almack, & Caswell, 2021) | **Practical and emotional issues**  ***Managing sudden change in condition and coordinating care***  *His condition became worse. I didn’t know what was going on. I’ve been managing him at home, but what happened about two weeks ago, I was frightened to death [he collapsed, and his eyes were staring at the sky]. (Eno, Mike’s wife)*  *He ‘dies’ and ‘resurrects’ most of the time. His condition has thrown us [the family] into a state of confusion. We don’t know what to do. (Sophia, Samson’s caregiver)*  *It’s terrifying. I feel like dying. I am. I don’t know how my condition is going to end. (Mike, patient)*  *. . .it’s not comfortable doing that all by myself. I need to ensure that I direct the affairs relating to care to avoid confusion and all that [addressing different duties all at the same time]. The task is not as simple as that. (Samuel, Tawiah’s son)*  ***Conflict in care provision***  *Some were pushing him to undergo chemotherapy while others vehemently opposed to it. Therefore, we are divided about what to do, and the other faction (who believe they use herbal medicines) are not happy and not helping with his care. (Kwaku’s wife, Okonore)*  *Our main problem is that he doesn’t eat enough. He needs to eat to get active and for us, too, to get the appetite to eat. If he refuses food for days, everyone is bothered too. (Eno, Mike’s wife)*  ***Significance of providing food for patients***  *I am unsure if I have pissed him off or something. I don’t figure out why he refuses his best food. I can’t tell. He might be annoyed, or he is doing that intentionally to end his life. (Sabi, Boat’s brother)*  **Navigating care at home**  ***Getting on with care (trial and error)***  *It’s mostly trying one thing or the other to see which one works best. We do ‘trial and error’ most times honestly. We are on our own when we are at home. Healthcare is not my field of training; mine is in accounting, and I don’t know how to nurse big wounds. (Teiko, Norbert’s son)*  *I must be thankful to the doctors for their excellent work at the hospital and for helping us to live. I can’t thank my wife enough. My wife is my ‘doctor’ at home, ensuring that I get all the care I need. (Baabamu, patient)*  *He is very heavy for only one person to provide personal care. He has a big sore in the lower back. … It’s difficult for us (caregivers) to avoid this if we can’t anticipate and know what to do. (Mawuli, Efo’s grandson)*  *…the doctor me told I had infections in my penis and my sacrum. We don’t know how to prevent or treat this at home. (Babaamu, patient)*  *Anytime my husband is discharged from the hospital, we take charge at home doing everything; because no health staff has ever come home to assist or something. If my husband’s condition becomes poorer, we then send him [back] to the hospital for a few days for management. (Abiba, Maegyida’s wife)*  ***Reciprocity: a duty to fulfil***  *We want to show him love and support by giving back what he did for us when we were young and fragile. (Agyei, Gyasi’s son)*  *We are also men; a similar thing (prostate cancer) might happen to us. You can’t tell what will happen. Can you? (Karikari, Atta’s son)*  *He is my birth father. He refused to send me to school even though he had the means to do so. I have decided to teach him how it feels like when someone rescues you timely. If I think about those past events, I wouldn’t have been here to care for him. (Joobu, Asamoah’s son)*  *I must be with him, especially this time he needs me the most, for better or worse, and in sickness and life. If I abandon him, I know I have offended the God I serve. (Achiaa, Opanin’s wife)*  *I must care for him. God will not forgive me if we abandon him because he (dad) has done a lot for the family and me. I can’t even stand the criticisms from others. Everyone knows what my dad has done for us growing up. (Sophia, Samson’s daughter)*  **Managing pain at home**  ***Assessing pain***  *It’s very severe pain, and I scream like a woman in labour (Boat).*  *Hmm, the pain is very unbearable sometimes. The last time I cried. That made my wife and children also followed suit as if we were mourning (Nobert).*  *You know, it’s hard. After giving all the medicines to him, and he still complains of pain. When it happens like that, I am entirely at a loss (Ivy, Nii’s daughter).*  *After I went for the procedure, when I came home that night, I couldn’t sleep at all. I was awake all night. I rested a while in the morning, and the pain came again. I complained. I asked for stronger pain relief, but it was ignored. Yes, it was. They (caregivers) think I might be demanding unduly (Nelson, patient).*  ***Access to pain medications***  *I prefer to get the medicines at the hospital where I go for review (tertiary hospital) because I am guaranteed of its effectiveness. But sometimes, they too, they run out of stock. (Ali, patient)*  *It has been a vigil night for us for the past few days, and we all were panicking. I didn’t know what to do after he complains even after giving him all his pain medicines [Tears flowing]. (Safia, Boat’s caregiver)*  ***Use of herbal medicines***  *We got some herbal medicines from a woman who is known to be an expert in that field. (Eno, Mike’s wife).*  *I went to (name withheld), and I got those medicines because they said when I use them, I wouldn’t need to have the catheter in place. I paid a lot. After some time, my problem became worse (had sores at the anus). I went there again, and still, my problem wasn’t solved. I had to stop and report to the hospital (Ofori, patient)*  *I took some herbal medicines (forgotten the name) to treat cancer. I stopped when I have sores in my anus (Opoku, patient).*  *I used some herbal medicines to treat my erectile problem. It was ok at first, but now the problem is worse (Maegyida, patient).* | Qualitative - High | Unchanged | Remains  unchanged | High | High: the dependability score is based on the specific questions from the critical appraisal scores for included studies related to the appropriateness of the conduct of the research with research aims and purpose 4-5 ‘yes’ responses, the paper remains unchanged  Remains unchanged as all findings  unequivocal |
| (Prado et al., 2022) | “**Floating between acceptance and resistance: perceiving death in a near horizon**”  *(PT_BHU_1) I was sad to know, because there are so many things that we would still like to do and cannot. But I have faith, the only one who knows things is God, and only He knows about tomorrow.*  *(PT_BHU_4) I don’t know for sure, I just know that it’s difficult, whatever God wants, I can’t really change much, so I left it up to God and them (health professionals), and then I think it will work.*  *(PT_BHU_2) the doctor said that this will not change and that it will not improve, but I think there is no way for him to guarantee that, it may be that with time it will improve.*  *(FC_BHU_3) I already knew deep down that she was in a very serious situation, but even so, our plan was to return home cured [* . . . *] but hearing that she would not get well, it took me off the ground, it was very difficult and still is.*  *(PT_BHU_5) when they told me that cancer had spread, it felt like I was regressing in life, I was doing so well, but at that moment it seems like it was all in vain, it shook me a lot.*  *(PT_PC_2) My life was getting back to normal, until the moment things got worse, and then everything changed, including her course, but what can you do, that’s what happens to those who are alive.*  *(PT_BHU_2) Knowing that [disease] was terrible, we don’t want to, we’re afraid, I didn’t want to go through what my sister-in-law went through, she suffered and had metastases too. They told me that my [cancer] is different, but it is happening too fast too, I didn’t expect it.*  *(FC_PC_3) She is elderly, so we already imagined that, but I don’t know if I’m ready for it [death] now, it’s been a different and very difficult experience, it’s the law of life [sighs] she’s suffering, not only physically, but mentally, she never wanted to be in this situation, maybe for her, it’s a relief.*  “**Analyzing the end from another perspective: it is in the encounter with death that life is perceived**”  (PT_PC_3) when I found out about my situation I cried a lot, I didn’t want to understand, I had only one thought. Now I have a new vision of things, I don’t have to keep thinking ahead, I say I’m just thinking about the now, the current moment.  (FC_PC_3) today we are much more realistic with the situation, it is heading towards the end, we are also going, we are all going to [die] . . . there are some people who are closer by nature, this is her case, for this is clear to me.  (FC_PC_2) I know it’s going to happen, but we don’t talk about it [death] we don’t like to talk, I realize that she gets a little weird when someone brings it up, and I also think it’s better not to talk, I know she’ll have it in her head all day, and it’s not good for her to dwell on it.  (FC_BHU_3) I even think about it [death] sometimes, but my experiences are not good, that’s why I don’t like it, it seems that everything comes to my mind again, I don’t want to spend it with her, so it’s not even good to think about it.  (PT_PC_5) it’s our time, but I prefer not to think about it [death] it seems that the more we think, the worse we get, it’s bad to keep it in our head all the time.  (PT_PC_3) Now I have a new view of things, before I wanted to be in public service, and have a good job so I could have a child. Nowadays I know I don’t need all that, I just know that we need to live one day at a time and think about this moment as if it were the last.  (PT_BHU_5) I usually say that cancer taught me many things, and one of them was this, the opportunity to stop a little, see things differently, today I pay attention to small details, before I lived a busy life, it was just working, today I know I may not have more time, so I want to enjoy it without worrying about looking at the clock.  “**Accepting the path: between the love of letting go and the love of wanting to stay**”  *(PT_PC_1) we talk about it [death] and I understand it well, I’m not afraid of dying anymore, however, what I think about most now is about her [daughter], because it’s just the two of us, I worry about leaving her alone.*  *(FC_PC_1) I was afraid of that moment [of death], but seeing her in bed, in this condition, it seems that she is no longer there, for me and it is sad, but it must be more painful for her, so I believe, that when she goes [to die], it’s going to be a rest, if she could talk, I’m sure she would want that too.*  *(PT_BHU_3) one day he will [die]* . . . *do what I don’t want is to suffer in a hospital bed, I don’t want that.*  *(PT_PC_5) I’ve been thinking about these things [death] and I only know one thing I don’t want, is to suffer in a bed giving them trouble [family].*  *(PT_BHU_2) My sister-in-law suffered a lot before she died, I don’t want to be like her, I already told her [daughter] that I want to go [die] before suffering.*  *(PT_PC_3) dying is a slow process, I gradually understood, today I can say that I am better prepared, I know there is nowhere to run, so it’s like I say: let’s live better, enjoy, and help others, while there’s still time.*  *(PT_BHU_5) [* . . .] *what bothers me is not death, it is knowing that I will no longer be here to do what I like, what I have now come to see, I joke that everyone had to experience cancer, just to have this feeling that tomorrow might not be here anymore.*  *(FC_PC_5) obviously, no son wants to say goodbye to his mother, for me he left her there in bed forever, but then I think, did she want that? I’m sure not. Still, I don’t know how I’ll react, if I knew when, I think I wouldn’t want to be there, even though I know it’s for the best.* | Qualitative - High | Unchanged | Remains  unchanged | High | High: the dependability score is based on the specific questions from the critical appraisal scores for included studies related to the appropriateness of the conduct of the research with research aims and purpose 4-5 ‘yes’ responses, the paper remains unchanged  Remains unchanged as all findings  unequivocal |
| (Ahmed et al., 2023) | **Care Coordination**  ***Communication between healthcare providers***  “*everybody is involved and everyone seems to know what’s going on- like all three branches- the RN who visits her at home or calls her, or the [Cancer Centre], or the GP. So that seems to be working well” (Caregiver 2, dyad)*  *“I think later on when we spoke with the palliative homecare team they would get in touch with the doctor- for instance to make something happen. That they knew medical oncologist would have the best chance at making it happen. They were able to intervene in the system and make something happen which we could never do. We were very grateful, so I guess it worked there for them to talk to one another.” (Caregiver 3, dyad)*  *“The oncologist asked me, that they have a nurse who deals with palliative care, and if she could visit me. And I agreed to that, and she told me when I last spoke to her, that she checks up about me with the oncologist, so they’re in contact.” (Patient 4)*  *“I’m glad that my doctor oncologist talked to my doctor and asked if he would be willing to look after some of the cancer- stuff and he said yes- he’d be more than happy to do it. So everybody really has been so good.” (Patient 5, dyad)*  *Suggestion: “Some way to represent how the services plug into each other and where the boundaries are of the services—cause that’s probably one thing that’s a little bit confusing. We would be offered a service from one part of the organization and it would be referred to another part ... full time caregiving at times is stressful, and these folks are busy so playing the telephone tag and messaging, it gets—it adds a little bit of stress to the home environment. There’s only so much you can remember as you get older.” (Caregiver 5)*  ***Role of Family Physician***  *“he’s been mum’s physician for a long, long time. So just having him support anything that’s going on is, you know... my mom’s very comfortable with him. And so, it just and it helps support the treatment he manages. You know, does the reorders for her steroids and that kind of stuff.” (Caregiver 1)*  *“_____ was happy to have the GP in her team because the GP was able to provide a far more general, holistic, common sense sort of perspective in the landscape as opposed to a specialist. You know we were grateful for the specialist but it was almost like a man with a hammer- who sees everything with a nail...GP that we had- excellent in that regard, great emotional support and a great help in providing comfort and advice on how to proceed and how to make a decision.” (Caregiver 3, dyad)*  *“yes he is he’s a longtime family doctor and has become a personal friend over the years and is very concerned about my case so yes he’s involved and informed, and I make sure that when they ask me about CT scans or MRIs—if I want any other doctor to look at them- I send them to him- because he phones me a lot to see how I’m doing... I know he thinks he let me down because we didn’t find it sooner, but it’s not his fault. That’s to say he’s very involved and concerned.” (Patient 7)*  *“he (FP) talks to me haha... and then he always goes over the results of any tests and that- and I mean the oncologist always goes over the tests from the scans and then he follows through and wants to know how I’m doing with this and that and the others, so he’s following. If I have any problems and ...if he doesn’t know the answer, he’ll get in touch with the oncologist.” (Patient 5, dyad)*  ***Relationship with Palliative Care Nurse***  *“I think my concerns- she takes note of it and she has told me personally*  *that she would pass on the information to the oncologist. And it’s been helpful too, when I had a fever and she came in to check on me, she directly called my doctors office....It puts my mind to rest I think, having that extra support because I’ve had to call the triage and so that takes hours for them to call back, and you know they’re busy so for a nurse to call back and that nurse that may be calling me back from the office, may have never seen me before. So I like the idea that I can call the palliative care nurse, and you know I can get a hold of them right away or they’ll call me back shortly thereafter.” (Patient 1)*  *“____ (PC nurse) is somebody that I’ve been so impressed with, and I really enjoy her, quite lovely, but also what she recommends and anytime she does make a recommendation it’s actually quite solid and down-to-earth and practical I really like that. And you know she just really wants to see how I’m doing and make sure that I’m right on track with what they’re offering me and it’s been working out really really well. She’s also a really great conduit to all the other people because you know if I have to go call the clinic, If I have to dial into clinic and get answers and help, It can always get into a giant game of telephone you know what I mean?” (Patient 6, dyad)*  *“she’s very experienced in what she does, you know quite organized...she came very well prepared, considering what the situation was and what we’re dealing with. And then just listened and asked some really probing questions about where we are at physically and psychologically, our home design, are we set up, and she had a bit of a checklist and pamphlet that gave us more information that we could follow- it wasn’t a one time sales pitch, it was delivered with kindness and empathy and well thought out conversation” (Caregiver 5)*  **Perception of Palliative Care & Advance Care Planning**  ***Timing of Palliative Care***  *“then more recently when we were put with the palliative homecare team,*  *then again made sense the timing was correct- xxx (patient) was starting to weaken and when it was suggested during a regular appt with the medical oncologist, everybody was in agreement that this was the right time to get back in touch with those folks to maybe look into some things to help us out. So I would say the timing is appropriate.” (Caregiver 3, dyad)*  *“I feel like it’s pretty good, I think the earlier the better. Even though there’s some stigma associated with it, if you’re talking to someone about palliative care that means you’re down the path along that end of life journey. For us, and I can’t speak for anyone else. For myself and my wife, the sooner we understood what was happening and what services were available, it gave us a piece of mind and it didn’t diminish the hope for best quality of life and what’s ahead. It did give us a chance to be a bit more prepared in our minds of what to expect and to know there’s services out there” (Caregiver 5)*  *“I think earlier on is better. Yeah, so that was right after the diagnosis that I had that it had spread right to my liver. It wasn’t stage 3 anymore, it was stage 4. So, at that point, and it was early on so that’s better so you kind of get accustomed to it. And earlier on I think is better than later.” (Patient 1)*  *“I don’t know how she could provide a key that I don’t need right? I could do everything myself right now, and I would not want to waste anyone’s time if they just come to check up on me. If I felt the need for a person, you know and I’m very grateful that there are people to help if you need the help, but I also feel that I don’t want to waste anyone’s time by... you know, feeling that they have to do anything for me when I don’t need it.” (Patient 4)*  ***Meaning of Palliative Care***  *“we had lots of discussions about this because mom was quite upset about being followed by palliative care. So, we certainly had the discussion that this is not end of life care, but this is more about controlling your symptoms. But palliative care claim to fame is to make people comfortable and more ethical with what we’re trying to do” (Caregiver 1)*  *“quality of life, there’s no remission- palliative, well you’re terminal not immediately necessary, you’re not at the end stage. It’s quality of life as disease progresses. So, it’s dynamic and not linear thing” (Caregiver 2, dyad)*  *“Mostly just supporting with symptoms so that the life you’re living is a good life...I did initially think end of life when I was referred to them, but they’ve since explained that it’s during life not just ending of life.” (Patient 2)*  *“palliative care is someone that comes in and helps you adjust to what you do living day to day and I know it can mean that you may have to go into palliative care somewhere. But I think its just the first step in looking after the treatment as it goes along.” (Patient 5, dyad)*  ***Advance Care Planning Discussions***  *“recently we changed those goals of care with the help and advice of the palliative care nurse we found that very helpful. Again they had a holistic landscape of the situation to help you make that decision. And the implications of those various choices. And they helped us to change the goals of care at the XXX (name) hospital” (Caregiver 3, dyad)*  *“At first I just looked at it, didn’t wanna do it. it took me time to accept as your mind kind of relaxes, and you think yea maybe we should plan for it. It’s going to happen so it’s good to be prepared. So yea I said I was going to take care of it, and gave my mom and dad the official sign, and put it in place” (Caregiver 4)*  *“She’s [PC nurse] very good at being- she’s frank but kind, and has no problem with having these kind of conversations whatsoever, she seems very good at it which is a huge comfort to have somebody been assigned to help you dance around the issue all the time and poking at it for both of us we’d rather have it on the table and be aware of what is available to us and what else we might need.” (Patient 6, dyad)*  *“it was tough, emotionally difficult, but was handled well and was handled in a way—she [PC nurse] said you don’t have to focus too too hard on it right now, but something to think about. There wasn’t too much pressure or anything.”(Patient 2)*  **Coping with Advanced Cancer**  ***Support from family and friends***  *“my parents have a lot of friends, they have a huge social*  *—it definitely made it easier for us, because they’ll come by and take my mom and dad out for like coffee. My mom loves ping pong and there’s a club where they all have their friends, so that’s where she’ll meet, and go and play some ping pong. If it was just the family, it would be really hard. So it’s nice to have friends who step up to the plate and make it easier” (Caregiver 4)*  *“a really good home caregiver is also a really good gate keeper. If you got somebody who can manage, and it could be a friend doing it too, it could be your spouse, but the gate keeper who says ‘she’s not having company today, she’s too tired’” (Patient 6, dyad)*  ***Support from healthcare system***  *“But for myself I could use a bit of support and it’s nothing to do with the cancer, but more so my relationship with my mom, so I don’t what they do there- I can find out by going, I’m sure I can find a counsellor” (Caregiver 2, dyad)*  *“She (oncologist) was accepting of the fact that xxx (patient) was talking to the herbalist- and was okay with that- as well as the naturopath who was giving her vitamin c- umm the herbalist xxx had been seeing for years and the naturopath xxx sought out and started to see. None of these were referrals from the conventional medicine world. What she did wind up with is someone from the conventional medicine world who was tolerant and sympathetic.” (Caregiver 3, dyad)*  *“when I was on the pills, I found quite reassuring that the pharmacist would call and see how you’re doing on the medication and give you some, you know, things weren’t working quite right? They would give me some ideas, and that’s very reassuring. In spite of having kids and friends and that, it’s a lone journey.” (Patient 5, dyad)*  *I met with her at the beginning just to make sure that my...financially I was able to handle this. And she supported with some of the paperwork required for my disability and had the doctor fill it out on my behalf and submitted it. It just took a little bit of the coordination away from me, which was helpful.” (Patient 2)*  ***Strength in faith***  *“I’ll also wish to mention here that I’m a bible believing Christian.Ilivemyentirelifebyfaith.FAITHnotFA T E. I don’t live my daily life by hope, I live my life by faith in the lord Jesus Christ. And it has been that way not since I’ve had cancer but from the very beginning of my life, I grew up in a Christian family and still do it that way.” (Patient 4)*  *“I just take it as it comes that way and I have a pretty strong faith...I go to church too that supports me as well.” (Patient 1)*  ***Value in Independence***  *Cooking, my wife and I do the cooking, the washing up, everything, I don’t feel that I am at that stage yet that I need any homecare, I do not need any. My wife and I do everything that needs to be done, without a struggle, or that it’s difficult for us to do- we just do it as we always have.” (Patient 4)*  *“xxx (patient) always took responsibility for all the decisions that were made uhh we both felt that because it’s her body and her life so it’s really her decision but I believe she was always grateful to have me present and attended any sessions of any significance” (Caregiver 5)*  **Patient and Family Engagement**  ***Taking initiative and being informed***  *“I probably ask way too much, I get copies of the blood*  *work...It’s almost like if you’re interested and take an interest in understanding the disease and symptoms and treatment management, then the doctors are really helpful. They know that you want to learn and understand and how you can be of help to them and the patient... I am, and also how available. I also think I’m very fortunate because I am retired and can spend that. I’m not sure what I would do if I was still working. We would have to have made some serious changes, and serious decisions.” (Caregiver 5)*  *“We had to make decisions and decide for ourselves whether things that were recommended to us were right or not and then if we felt that the option wasn’t right we always investigated further or figured out what to do ourselves. So it was important to us to have that advice and support” (Caregiver 3, dyad)*  ***Patient Advocacy***  *“You know if there was one thing that could be added on is the you know for somebody who doesn’t have that kind of support- some kind of patient advocate being assigned to somebody who doesn’t have that kind of support. I think it’s really important for them not to go to the appointment by themselves” (Caregiver 2, dyad)*  *“The patient is the quarterback. And whether we like it or not, we have to get educated on what’s going on because decisions will be made that we don’t understand or might not like.” (Caregiver 3, dyad)*  *“but the medical side, they were fighting me so that was tough it was very tough to stand up to that medical team when you know this is a very personal decision do you know what you do here in the circumstances and I said I know what I need to do and I’m doing it, but they really had a hard time with it really, really had a hard time with it.” (on choosing to stop chemo) (patient 6, dyad)* | Qualitative - High | Downgrade 1 level  – Moderate | Remains  unchanged | Moderate | Downgraded one level as  scored 2–3 out of 5 for questions relating to appropriateness of conduct of the research.  Remains unchanged as all findings  unequivocal |
| (Brose, Willis, & Morgan, 2023) | **The intentional pursuit of engagement in everyday activities**  ***Purposefully striving for independence***  *Interviewer: “Why is that important to you, to be independent?”*  *Lisa, Interview 1: “Because it’s everything.”*  *“For me, just to be able to do a couple of dishes, put my hands in the warm water, and to be able to do that function . . . I’m grateful for that at that point. . . . And just Swiffering, being grateful that I can just Swiffer a patch up. . . . I can go, yeah, that looks clean and sanitary, and I feel good about it” (Jessica Interview 1).*  *“Tonight I’m going to the school to watch my son in a play . . . [the] horrible bleachers [cause pain] but it is super fun, so that’s totally worth it. Something like that really is joyful”. (Interview 8).*  *“I’m able to do more. I’m able to get out more. I’m feeling better. I’m up all day and not sleeping all day. . . . I can turn my toothbrush off and on again, and I can brush my own hair! . . . [It makes me feel] really good, and I’m showering myself again. . . . I can shower and dry. Not do everything for dressing myself, but a lot of things. Gaining some of my independence back [albeit in modified form] feels really good!” (Interview 2).*  ***Prioritising what is important***  *“I want to give myself goals. I want to set achievements ahead and not just sit around and wait to die. . . . It just setting those goals and those milestones, it gives you a purpose” (Interview 1).*  *“It gives you a bit of a purpose. Plus, I like my job and I like the people I work with, so I’d rather be there than sitting around at home not feeling well or whatever” (Interview 1).*  ***Navigating relationships***  *“It’s just being there for them. Honestly, that’s the most important thing, and being able to answer their questions and just to be their dad. . . . Your outlook on life changes, so it’s those little things that matter the most. The sit down at the dinner table and being able to talk about the day. Before, it was just kind of eh, it’s part of the day, but it means a lot now” (Interview 1).*  *“Either they see you as strong, or they just want to fix it, so you don’t want to talk to them about it, or they think you should be positive all the time, so they get mad when you say something negative because they feel like you’re not going to get better if you’re not positive” (Interview 5).*  **The challenge of unrelenting change and loss**  ***Living with unrelenting change and uncertainty***  *“[Cancer] changes everything about your life and how you are in the world, who you are, and that there is nothing in life that it doesn’t touch… Even though you’re the same person, it changes and touches every aspect of your life.. I think it changes the way people see you, and it changes your priorities, it changes your finances, it changes your career life, . . . it changes the relationships with everyone around you because they see you differently and you see yourself differently, . . . it changes your physical abilities, your mental capabilities… I feel like it affects every part of your life” (Melissa Interview 10).*  ***Losing independence as I become unwell.***  *“You just want to go back to the way it was, and part of that is going to work. When I was first diagnosed, it was, yeah, I want to go back to work, I’ll be back after spring break. Then it was really quickly apparent that that wasn’t going to happen. I was like well back after the summer. Then it was it was like well no you won’t. Now I’m kind of at peace that I won’t go back to work” (Interview 7).*  **Adapting to change is an active, ongoing process**  ***I need to adapt to keep doing what I want to do***  *Tammy wore loose clothing to dress indepen- dently despite minimal hand function (Interview 1).*  *Jessica used the dishwasher when handwashing dishes became too tiring (Interview 1) and David began to use an electric razor instead of a blade to shave (Interview 4).*  *When Amanda was unable to take her dog for a walk, she found ‘other ways to do things together, cuddle up on the couch and watch a movie’ (Interview 1).*  *David shifted from in-person shopping to online browsing, ‘seeing the different things, the new plumbing stuff. . .now I go on YouTube and look at the new gadgets’ (Interview 4) whilst Tammy shifted from independently donning a swimsuit to requiring assistance from others in order to continue swimming (Interview 5).*  *Tammy became excited about the little things, such as being able to ‘pluck the chin hairs out of my chin’ (Interview 8). This also helped participants adjust to their increasing dependence, such as Peter describ- ing how he tried ‘to make the most of it and look on the positive side of things’ when having to adapt how he played with his kids (Interview 1).*  *“Just getting out for a short walk versus doing a big hike. It’s still I’m getting out, I can be in nature a little bit, but it’s not huge. [My husband] takes me for drives. I like driving, but if I can be the passenger and still see things just as we drive around, I enjoy that. It’s not full-on getting out there. Around here, there are so many scenic drives, like we’re kind of lucky. Yeah, I’m not hiking, I’m not in the backcountry, but I’m still seeing beautiful scenery and spending time with him because he never liked hiking, so it works. We call it going for a walk in the car” (Interview 10).*  ***I need to keep adjusting how I do things as I get sicker***  Melissa described how she had adjusted how she structured her day in order to do what was important to her:  *“Ideally, I would structure the day to do a couple things in the morning, have a little lie down in the afternoon, maybe do one more thing, and then go to bed early and stay in bed longer in the morning too. I could live with that, if doing that wouldn’t be too painful the next day.”* | Qualitative - High | Downgrade 1 level  – Moderate | Remains  unchanged | Moderate | Downgraded one level as  scored 2–3 out of 5 for questions relating to appropriateness of conduct of the research.  Remains unchanged as all findings  unequivocal |
| (Pérez Sandoval et al., 2023) | **Creencias para mantener la poca salud y aliviar síntomas**  ***Bondades de la medicina homeopática***  *“Me recomendaron tomar unas gotas de essências florales que me ayudan a mantenerme tranquila” (1E).*  ***Creencias acerca del final de la vida***  *“No pues que puedo decir...eso ni lo he pensado, solo sé que el día que Dios diga, esto hasta aquí, ese día me voy, pero por ahora estoy contento” (7E).*  *“Yo pienso que a todo el mundo le toca llegar al final de la vida, lo que no sabe uno es cuándo” (4E).*  ***Medicina convencional o alopática***  *“La única forma de tratar mi cáncer a nivel científico, es por medio de la quimioterapia, además de las terapias de quimioterapia no he hecho más nada” (8E).*  ***Remedios naturales que curan el cáncer***  *“La bebida de la vida: es un licuado con moras, remolacha, bueno con muchas cosas de frutos rojos, si incluso le colocan cosas como hígados y mollejas, de pollo también”(1E).*  *Además, algunos participantes refirieron que la enfermedad y algunos tratamientos les generaban síntomas como náuseas, vómito, distensión abdominal; por lo tanto, el consumo de plantas como: tallo de rama de apio, hierbabuena, albahaca y manzanilla les mejoraron estos síntomas: “Manzanilla, albahaca y una rama de apio para cuando uno tiene el estómago inflamado” (9E)*  *“Infusiones de hojas de guanábana, también le hecho una hojita de kalanchoe, un limón congelado rayadito y también sábila” (1E)*  ***Espiritualidad/Religiosidad***  *“Pegado al señor y a la santísima virgen María, totalmente, yo soy entregado a él” (4E). Consideraronla oración como opción para mejorar los síntomas generados por la enfermedad. “Toda gira alrededor del señor, de mi esperanza, de mi confianza en él, la misericordia; él me va a sanar y voy a quedar libre del cáncer” (1E).*  **Prácticas culturales**  ***Búsqueda de información en la red***  *“Encontré un chico en internet que decía que para el tumor era bueno tomar agüita de llantén” (2E). “Utilizaba computador cuando me servía, pero ahora para cualquier cosa tengo un buen celular para averiguar todo por este medio.” (1E)*  ***Consumo de alimentos y preparados “anticancerígenos”***  *“Tomar agüita de llantén, infusiones de hojas de guanábana, un licuado de remolacha, manzana, una hojita de Kalanchoe, un limón, también sábila”(1E).*  *“Hojitas guanábana, la tomaba me hacía esa agüita. “El agua de anamú, la hoja de guanábano, agua de yerbabuena, agua de manzanilla” (2E).*  ***Prácticas espirituales/religiosas***  *“Voy a la iglesia y acá en la casa me la paso orando, siempre en la mente y en mi corazón está Dios, por encima de Todo” (2E)*  *“Orar y hablarle, hablarle frente a frente al mismo señor que está en todas partes, osea está en todas partes vivo escuchándolo a uno” (3E)*  ***Mezcla de Tratamiento Complementario y convencional***  *“Mi mamá también está tomando las pastillas de transfer factor de 4life y el riovida, mi médico dice: tómese lo que quiera, igual eso no le va a servir, es la Fé que usted le ponga”..(2E)*  *Algunos productos de manejo suplementario no tienen suficiente evidencia científica, por ejemplo, existen prácticas como el consumo de productos químicos como el dióxido de cloro, que pudieran ser perjudiciales, pero ante la esperanza de alivio de síntomas y la curación, los participantes asumen los riesgos: “Un señor me regaló una botellita de dióxido de cloro y me tomó un centímetro en un vaso de agua, lo puedo tomar dos veces al día o tres veces tiene un saborcito como a límpido o a clorox, espero me ayude”.. (2E)*  *“Veneno de escorpión azul, es un remedio cubano, los médicos de allí, revisan tu historia y te explican la forma de cómo se prepara, cada cuanto lo tiene que tomar y qué cantidad” (5E)*  **Experiencias en la adaptación a la enfermedad y muerte**  ***Apoyo Familiar***  *“De todos mis hijos tengo apoyo, están muy pendientes de mí” . “Sobre todo mi mamá es mi mano derecha, es quien ha estado pendiente de mí y tengo un hermano, es un apoyo” ) (8E)*  *“Recibí mucho apoyo y recomendaciones de todo el mundo, porque sale mucha gente a decir cosas sobre la enfermedad”..(4E)*  *El acompañamiento que reciben por parte de familiares y cuidadores, en el proceso de enfermedad, está centrado en todo tipo de atenciones, entre ellas se encuentra el hecho de preparar y darles “remedios caseros “que están basados en sus experiencias y conocimientos: “Mi mamá me dice que el jugo de frutos rojos como el agraz y la mora me sirven para subir mis defensas” (8E)*  ***Cambios en los hábitos***  *“Ahora soy más consciente de mi alimentación como más pescado, como más carne blanca” “No como carnes rojas, lácteos, dulces..(5E)*  *Por otra parte, la trayectoria de la enfermedad, va dejando cambios en el estado funcional de las personas: “Claro, yo era deportista, salía a correr, brincaba, hacía muchas cosas, salía a jugar fútbol y ahora pues le cambia a uno la vida, ya no soy capaz de nada” (7E)*  *“Ya no puedo hacer tanto, llevo como dos meses sin poder salir a la calle como solía hacer antes” (8E)*  ***Conocimiento de la enfermedad***  *“El cáncer es una enfermedad, células que no salen del cuerpo más que se van multiplicando, el primer médico que descubrió, lo llamó así porque parecía un cangrejo” (1E)*  ***Programa de Cuidados paliativos domiciliarios***  *“Los cuidados paliativos son los que se le dan a una persona para mejorar su calidad de vida, un estado en donde se sienta estable, tranquilo, alegre, donde intervienen un psicólogo, ayuda espiritual, los terapistas respiratorios es un grupo, ellos me han dado el apoyo” (7E)*  *“Ellos están pendientes de tanto física, psicológica y hasta espiritual, de la Fé del paciente y de cómo vive en el entorno familiar, cómo lo cuidan las personas que están con uno, la psicóloga, la trabajadora social, no tenía idea existiera esa forma hasta que me lo formularon y me remitieron de cuidados paliativos” (1E).* | Qualitative - High | Downgrade 1 level  – Moderate | Remains  unchanged | Moderate | Downgraded one level as  scored 2–3 out of 5 for questions relating to appropriateness of conduct of the research.  Remains unchanged as all findings  unequivocal |

Note: High (dependability): the dependability score of more than 50% of the contributing studies is 4 or 5. High (credibility): the synthesised finding contains only unequivocal findings. Downgrade 1 level: the synthesised finding contains a mixture of unequivocal and equivocal findings.

**Facilitating experiences of people in palliative situations at home**

| Personal: Feelings | Hope and Belief in the Future  *... but I shall go on fighting ... hm ... I have a feeling that my life is not finished yet ...*  *I think that ... It can be painful and hard with the pain ...but I hope I do not have to be alone at the time...*  *I am aware that it doesn’t cure but I hope it can bring relief ... so it’s a hopeful feeling anyway.*  Safe but Unsafe at Home  *... It’s easier to be unwell if I can stay at home ...*  *we embrace each other frequently and a great deal and it feels safe and secure ...*  *... we are able to be together and it makes us happy in the midst of the sorrow ...*  Better to Manage Care at Home  I *only get reminded of a lot of negative things by visiting the hospital. For me it’s just, if I go to the hospital, I have to recover during the following 24 hours, it is nothing but negative...it’s much better if everything could be managed here (at home).* |
| --- | --- |
| Personal: Spirituality and faith | “Floating between acceptance and resistance: perceiving death in a near horizon”  *(PT_BHU_1) I was sad to know, because there are so many things that we would still like to do and cannot. But I have faith, the only one who knows things is God, and only He knows about tomorrow.*  *(PT_BHU_4) I don’t know for sure, I just know that it’s difficult, whatever God wants, I can’t really change much, so I left it up to God and them (health professionals), and then I think it will work.*  *(PT_BHU_2) the doctor said that this will not change and that it will not improve, but I think there is no way for him to guarantee that, it may be that with time it will improve.*  *(FC_BHU_3) I already knew deep down that she was in a very serious situation, but even so, our plan was to return home cured [* . . . *] but hearing that she would not get well, it took me off the ground, it was very difficult and still is.*  *(PT_BHU_5) when they told me that cancer had spread, it felt like I was regressing in life, I was doing so well, but at that moment it seems like it was all in vain, it shook me a lot.*  *(PT_PC_2) My life was getting back to normal, until the moment things got worse, and then everything changed, including her course, but what can you do, that’s what happens to those who are alive.*  *(PT_BHU_2) Knowing that [disease] was terrible, we don’t want to, we’re afraid, I didn’t want to go through what my sister-in-law went through, she suffered and had metastases too. They told me that my [cancer] is different, but it is happening too fast too, I didn’t expect it.*  *(FC_PC_3) She is elderly, so we already imagined that, but I don’t know if I’m ready for it [death] now, it’s been a different and very difficult experience, it’s the law of life [sighs] she’s suffering, not only physically, but mentally, she never wanted to be in this situation, maybe for her, it’s a relief.*  “Analyzing the end from another perspective: it is in the encounter with death that life is perceived”  (PT_PC_3) when I found out about my situation I cried a lot, I didn’t want to understand, I had only one thought. Now I have a new vision of things, I don’t have to keep thinking ahead, I say I’m just thinking about the now, the current moment.  (FC_PC_3) today we are much more realistic with the situation, it is heading towards the end, we are also going, we are all going to [die] . . . there are some people who are closer by nature, this is her case, for this is clear to me.  (FC_PC_2) I know it’s going to happen, but we don’t talk about it [death] we don’t like to talk, I realize that she gets a little weird when someone brings it up, and I also think it’s better not to talk, I know she’ll have it in her head all day, and it’s not good for her to dwell on it.  (FC_BHU_3) I even think about it [death] sometimes, but my experiences are not good, that’s why I don’t like it, it seems that everything comes to my mind again, I don’t want to spend it with her, so it’s not even good to think about it.  (PT_PC_5) it’s our time, but I prefer not to think about it [death] it seems that the more we think, the worse we get, it’s bad to keep it in our head all the time.  (PT_PC_3) Now I have a new view of things, before I wanted to be in public service, and have a good job so I could have a child. Nowadays I know I don’t need all that, I just know that we need to live one day at a time and think about this moment as if it were the last.  (PT_BHU_5) I usually say that cancer taught me many things, and one of them was this, the opportunity to stop a little, see things differently, today I pay attention to small details, before I lived a busy life, it was just working, today I know I may not have more time, so I want to enjoy it without worrying about looking at the clock.  “Accepting the path: between the love of letting go and the love of wanting to stay.”  *(PT_PC_1) we talk about it [death] and I understand it well, I’m not afraid of dying anymore, however, what I think about most now is about her [daughter], because it’s just the two of us, I worry about leaving her alone.*  *(FC_PC_1) I was afraid of that moment [of death], but seeing her in bed, in this condition, it seems that she is no longer there, for me and it is sad, but it must be more painful for her, so I believe, that when she goes [to die], it’s going to be a rest, if she could talk, I’m sure she would want that too.*  *(PT_BHU_3) one day he will [die]* . . . *do what I don’t want is to suffer in a hospital bed, I don’t want that.*  *(PT_PC_5) I’ve been thinking about these things [death] and I only know one thing I don’t want, is to suffer in a bed giving them trouble [family].*  *(PT_BHU_2) My sister-in-law suffered a lot before she died, I don’t want to be like her, I already told her [daughter] that I want to go [die] before suffering.*  *(PT_PC_3) dying is a slow process, I gradually understood, today I can say that I am better prepared, I know there is nowhere to run, so it’s like I say: let’s live better, enjoy, and help others, while there’s still time.*  *(PT_BHU_5) […] what bothers me is not death, it is knowing that I will no longer be here to do what I like, what I have now come to see, I joke that everyone had to experience cancer, just to have this feeling that tomorrow might not be here anymore.*  *(FC_PC_5) obviously, no son wants to say goodbye to his mother, for me he left her there in bed forever, but then I think, did she want that? I’m sure not. Still, I don’t know how I’ll react, if I knew when, I think I wouldn’t want to be there, even though I know it’s for the best.*  Spiritual need  *I still do light housework, such as washing dishes and sweeping floors. Here, I think everything is still as usual.... (Participant 1).*  *When I got along with my friends, I did not mention my illness to “save face”, and they pretended not to know I was sick. It’s good for us (Participant 5).*  *I am very worried about my wife’s life after my death. She is a sensitive and fragile woman (Participant 1).*  *I want to alleviate the pain and prolong my life. I seldom think of the prognosis or death (Participant 6).*  *I suffer extreme pain every minute. I want to end my pain with euthanasia (Participant 15).* |
| Personal: Stable socioeconomic status | Enablers and Disablers: Financial Resources  *I think the commode chair, we had it for 28 days. That will be ending this week, and they want a ridiculous price, like $50 a month to rent. . .we’re going to have to figure out what we’re going to do. (Caregiver 74M)*  *I have to budget too...Because every week I get somebody for Saturday and Sunday. . .So I allocate four weekends so I need at least $400 and then the rest I will allocate it for the diapers and all that she needs...It’s a lot to manage. (Caregiver 69F)*  Use of herbal medicines  *We got some herbal medicines from a woman who is known to be an expert in that field. (Eno, Mike’s wife).*  *I went to (name withheld), and I got those medicines because they said when I use them, I wouldn’t need to have the catheter in place. I paid a lot. After some time, my problem became worse (had sores at the anus). I went there again, and still, my problem wasn’t solved. I had to stop and report to the hospital (Ofori, patient)*  *I took some herbal medicines (forgotten the name) to treat cancer. I stopped when I have sores in my anus (Opoku, patient).*  *I used some herbal medicines to treat my erectile problem. It was ok at first, but now the problem is worse (Maegyida, patient).* |
| Personal: preparation and knowledge | Caring actions  *´When he had the catheter, I would administer the medicine, but a few times he would do it, I help him with the curing procedures, all that, help him to vacate the colostomy and those things I have already learnt how to manage´.*  *C4E1*  Expected responses  *‘I’d say they should offer him support, to indulge him more, to help me, and keep us company’.*  *C4E4*  *‘I still need to know how to manage those things, I have to pay attention. Although he also pays attention and he knows how his things are applied, the colostomy and all also, he has learnt some things’.*  *C5E5*  Physical need  *I feel so much pain every day. I couldn’t have a rest for a long time because of the pain (Participant 12). After the activity, I often feel very painful, but I try my best to endure pain due to fear of drug addiction (Participant 14).*  *My family unanimously decided to insist on antitumor therapy. I wish to alleviate the pain and prolong life as long as possible (Participant 10).*  Enablers and Disablers: *Caregiver’s Role*  *No, he [husband] handles.... Without him I’d be dead. (Patient 77F)*  *“I’ve had to do the scheduling. I’ve had to deal with the [personal support worker], deal with the nursing, deal with what he needs for equipment” (Caregiver 75F).*  *‘The oxygen stops a lot, they ordered medications, we were discharged and at this time we have not gotten authorizations for any of them, not even morphine, nothing’.*  *C1E1*  Practical and emotional issues: *Managing sudden change in condition and coordinating care*  *His condition became worse. I didn’t know what was going on. I’ve been managing him at home, but what happened about two weeks ago, I was frightened to death [he collapsed, and his eyes were staring at the sky]. (Eno, Mike’s wife)*  *He ‘dies’ and ‘resurrects’ most of the time. His condition has thrown us [the family] into a state of confusion. We don’t know what to do. (Sophia, Samson’s caregiver)*  *It’s terrifying. I feel like dying. I am. I don’t know how my condition is going to end. (Mike, patient)*  *. . .it’s not comfortable doing that all by myself. I need to ensure that I direct the affairs relating to care to avoid confusion and all that [addressing different duties all at the same time]. The task is not as simple as that. (Samuel, Tawiah’s son)* |
| Community: Support from family and friends | Clinging to current daily life  *I am grateful that my children, daughter-in-law, and day service center staff kindly take turns caring for me.*  *I want to stay in the house where my husband and I built a life together despite the fact that he was rigid. (Eighty-seven, female, others)*  *I would be happy if my son and his family came back to live with me in the future. (Eighty-three, female, one-person household)*  *I want to stay in the house where my husband and I built a life together despite the fact that he was rigid. (Eighty-eight, female, others)*  *I want to stay in the house that I built until the very end of my life. (Eighty-seven, male, others)*  Enablers and Disablers: Community Support  *“was happy to be with them and have a chat with them…we get those emotional support from our parish community” (Caregiver 69F).*  Navigating care at home: Reciprocity: a duty to fulfil  *We want to show him love and support by giving back what he did for us when we were young and fragile. (Agyei, Gyasi’s son)*  *We are also men; a similar thing (prostate cancer) might happen to us. You can’t tell what will happen. Can you? (Karikari, Atta’s son)*  *He is my birth father. He refused to send me to school even though he had the means to do so. I have decided to teach him how it feels like when someone rescues you timely. If I think about those past events, I wouldn’t have been here to care for him. (Joobu, Asamoah’s son)*  *I must be with him, especially this time he needs me the most, for better or worse, and in sickness and life. If I abandon him, I know I have offended the God I serve. (Achiaa, Opanin’s wife)*  *I must care for him. God will not forgive me if we abandon him because he (dad) has done a lot for the family and me. I can’t even stand the criticisms from others. Everyone knows what my dad has done for us growing up. (Sophia, Samson’s daughter)* |
| Social: Support from healthcare professionals | See the Person  *And I actually discussed the matter with the doctor here, we sat down and talked about it for almost an hour, about how things would turn out and how it (dying) would be. She said, “We are here for you all the way.” “All the way,” now I know exactly what they mean.*  Practical Needs: *Transportation*  *Well, a lot of those ambulance people, they are quite something. Quite organized and better than what they used to be because you’re not doing well if you don’t know when if you’re coming back home you’ve got the voyager chairs . . .stretcher as well as a chair if you’re able to sit. And I thought that was quite interesting because I’ve never been in one before that. I’ll say where’d you get that chair, I can use that at home. (Patient 82F)*  Practical Needs: Healthcare Providers in the Home  *It won’t be care in the sense of hands on care. He [physician] won’t be changing diapers or linen... I think he’ll be monitoring the effects of his medication, his vital signs and he’ll probably have a baseline of his cognitive ability. So, he’ll know if it’s going down. I hope the physician comes at least once but is available if I need to talk. (Caregiver 82F)*  *“If there are any concerns, we just put a call through and. . . he’ll either provide a solution over the phone or he’ll say I’ll be out in 15 minutes” (Caregiver 74M).*  *I know any nursing care I need, if I need that drain- age tube drained, they’ll come in and do that, they’ll change bandages. . . I know helping with dressing, helping with baths if I need it.* (Patient 70F)  Enablers and Disablers: Education  *I had to have a plan. . .because they were coming in and out of the hospital room 13 times [a day]. . . so the pharmacist came and he gave me a plan of when to give drugs and how much. . . once I got home, the nurse helped me scale it down to like six times, and the doctor helped too... I can’t imagine having to do 13 or 14 different things in a day. (Caregiver 64F)*  *No…Because I can't evaluate his condition. So if his condition were to deteriorate and really required a hospitalization, I'm not in a position to make that call. (Caregiver 63F)* |

**Difficult experiences of people in palliative situations at home**

| Personal: Feelings | A Sense of Powerlessness  *I have asked once in a while if they can do anything ... but they did not answer me really ...*  Essential Meaning - Uncertain Safety  *I have asked once in a while if they can do anything ... but they did not answer me really ...*  Anxiety about the future  *I want to remain at home as long as possible and avoid being hospitalized. (Sixty-seven, female, others)*  *I really dislike hospitals. (Eighty-three, male, others)*  *Loneliness is unavoidable as we grow older.* *(Seventy-eight, female, one-person household)*  *I cannot ask my son for care support because I do not feel at ease with my daughter-in-law.* *(Eighty-seven, female, others)*  If I develop severe dementia, I would like to be institutionalized to avoid becoming a burden on my wife. *(Seventy-two, male, others)*  *I worry that I may not be able to discuss care services with the hospital or care service center if my condition deteriorates. (Seventy-five, male, one-person household)*  Abandonment of control  *I think I would have no choice but to be institutionalized in the future, just as my sister was. (Eighty-seven, male, others)*  *I do not know what I am going to do until I am in that situation. (Seventy-five, female, one-person household)*  *I do not want to think about it for now. (Seventy-five, female, one-person household)*  Delegating decision-making  *Because I live alone and have financial difficulties, I want to leave a power of attorney to my children. (Seventy-eight, female, one person household)*  *I have never thought about it, but I hope my brother will take care of me in the last days of my life. (Seventy-four male, one-person household) (Seventy-four male, one person household)*  Life changes  *‘It is hard work, especially the way we have to do it’.* *C1E1*  Coping  *‘For her it has been difficult, difficult to wear a nappy and it is even more difficult for me; she is my mother’.*  *C3E1*  *‘I don’t understand why didn’t they do the chemo, why didn’t they do chemo, that is what I wonder every day, why? Why?’*  *C3E1*  Psychological experience  *I often forgot and didn’t realize I was a patient. When my peer was upset and sad, I would enlighten them and make them happy and relaxed (Participant 5).*  *When I was diagnosed with lung cancer, I couldn’t ac- cept it at that time. Now my mood is relatively calm, because I have no choice but to accept it (Participant 11). There is no use crying over the illness. I pretend to be optimistic with my family. Actually, I am deeply anxious and sad every day (Participant 12).*  Significance of providing food for patients  *I am unsure if I have pissed him off or something. I don’t figure out why he refuses his best food. I can’t tell. He might be annoyed, or he is doing that intentionally to end his life. (Sabi, Boat’s brother)* |
| --- | --- |
| Personal: Self-care deficit | Change of Everyday Life  *... I want to live as usual but it’s not possible ... I can’t cook ... I can’t go for a walk ...”*  *it’s not a good feeling that she has got both our jobs now*  *...there are a lot of things which could be planed more calmly, but ...*  *...when you are healthy it is ... not your way of thinking I’m afraid ... you take everything for granted.*  Emotional and physical burden  *‘I am filled with angst, when he says this or that ...’* *C3E3*  *‘That makes more at ease and, well, I know that with the few people that I can count on in my family, we support each other, so I know he will be well, that is what is important’.*  *C1E1*  Health and Well-Being  *Some patients’ health was worse than anticipated once they arrived home; one expressed he did not expect to “experience so much fatigue” (Patient 62M).*  *Several participants expressed feeling secure in the hospital because of access to continuous nursing care and therefore felt “apprehensive” (Patient 48F) about going home: “I’m scared because I’m not going to have somebody [in the home] 24/7. . . what if I fall, if somebody’s not there. I’d get hurt” (Patient 51F).*  *“After a couple days [I realized] this ain’t so bad****. I*** *can manage here by myself.”* *(Patient 51F).*  Managing pain at home: Assessing pain  *It’s very severe pain, and I scream like a woman in labour (Boat).*  *Hmm, the pain is very unbearable sometimes. The last time I cried. That made my wife and children also followed suit as if we were mourning (Nobert).*  *You know, it’s hard. After giving all the medicines to him, and he still complains of pain. When it happens like that, I am entirely at a loss (Ivy, Nii’s daughter).*  *After I went for the procedure, when I came home that night, I couldn’t sleep at all. I was awake all night. I rested a while in the morning, and the pain came again. I complained. I asked for stronger pain relief, but it was ignored. Yes, it was. They (caregivers) think I might be demanding unduly (Nelson, patient).* |
| Personal: Low socioeconomic status | Social support  *Once I attended a classmate’s wedding, and some peo- ple were afraid to have dinner with me due to fears of cancer contagion (Participant 13).*  *When I passed them (my friends), they pretended not to see me. They worried I would ask them for money. Actually, I didn’t (Participant 11).*  *Over the past three years, I had received more than 20 chemotherapy treatments. The expensive medical bur- den forced us to sell our only house and borrow money from our relatives and friends, racking up medical bills of more than 1 million RMB (Participant 6).*  *I lived in a rural area, and the rural new cooperative medical scheme could reimburse only 30%. The reimbursement rate was too low to maintain anticancer therapy (Participant 9).* |
| Personal: lack of preparation and knowledge | Informational need  *After six months of treatment, the condition was not getting better. I begged and urged my wife to tell me the truth (Participant 7).*  *My wife replaced a colostomy bag for me yesterday. I found a macerated peristomal site with surrounding excrement. We had to go back to the hospital (Participant 15).*  Physical discomfort and pain  *‘The pain seems worse, yes, because it often does not stop – not even at night. Perhaps only once it would cease but the rest of the time it would not, at dawn the pain would calm down for some time and he was well without pain, but it would return again’.*  *C4E4*  *‘Lots of pain, especially in the lower limbs”.*  *C4E4*  Practical needs: Setting Up the Home for Care  *I thought we would be okay, but it’s only when he got home, and he had to lie on this chesterfield which is narrow. Then I recognized that things are different and that I need to rearrange everything in the room, get more equipment. I wish I had known that before. (Caregiver 82F)*  Enablers and Disablers: Uncertainty  *“I didn’t know anything and I wasn’t being taught any- thing on how to handle my own condition. You know, so it’s a little scary” (Patient 77F).*  *Things are always clear until you have to do it your- self. If she’s in pain...what do you do? And how quickly are you allowed to give the breakthrough medication? And are there limits to this if she’s feel- ing more pain than before? (Caregiver 48M)*  Practical and emotional issues: Conflict in care provision  *Some were pushing him to undergo chemotherapy while others vehemently opposed to it. Therefore, we are divided about what to do, and the other faction (who believe they use herbal medicines) are not happy and not helping with his care. (Kwaku’s wife, Okonore)*  *Our main problem is that he doesn’t eat enough. He needs to eat to get active and for us, too, to get the appetite to eat. If he refuses food for days, everyone is bothered too. (Eno, Mike’s wife)*  Navigating care at home: *Getting on with care (trial and error)*  *It’s mostly trying one thing or the other to see which one works best. We do ‘trial and error’ most times honestly. We are on our own when we are at home. Healthcare is not my field of training; mine is in accounting, and I don’t know how to nurse big wounds. (Teiko, Norbert’s son)*  *I must be thankful to the doctors for their excellent work at the hospital and for helping us to live. I can’t thank my wife enough. My wife is my ‘doctor’ at home, ensuring that I get all the care I need. (Baabamu, patient)*  *He is very heavy for only one person to provide personal care. He has a big sore in the lower back. . . . .. It’s difficult for us (caregivers) to avoid this if we can’t anticipate and know what to do. (Mawuli, Efo’s grandson)*  *. . . .. the doctor me told I had infections in my penis and my sacrum. We don’t know how to prevent or treat this at home. (Babaamu, patient)*  *Anytime my husband is discharged from the hospital, we take charge at home doing everything; because no health staff has ever come home to assist or something. If my husband’s condition becomes poorer, we then send him [back] to the hospital for a few days for management. (Abiba, Maegyida’s wife)*  Managing pain at home: Access to pain medications  *I prefer to get the medicines at the hospital where I go for review (tertiary hospital) because I am guaranteed of its effectiveness. But sometimes, they too, they run out of stock. (Ali, patient)*  *It has been a vigil night for us for the past few days, and we all were panicking. I didn’t know what to do after he complains even after giving him all his pain medicines [Tears flowing]. (Safia, Boat’s caregiver)* |
| Community: Lack of family support | Bond between the individual in palliative care and their caregiver (dyad)  *‘My mother sometimes thinks I scold her because I tell her to be patient, but I tell her: “mom let’s not get too anxious”’. C2E2*  *‘I think my mother wants to die at home’.*  *C3E3* |
| Social: Difficult access to health systems | Social support  Practical and emotional issues (*Managing sudden change in condition and coordinating care, Conflict in care provision, Significance of providing food for patients)*  Precarious mutual support  *I want to stay with my wife and care for her even though she has dementia. (Eighty-one, male, older couple only household)*  *I have to go on with daily life and continue caring for my wife despite my worries about her future. (Seventy, female, older couple only household)*  Expected responses  *‘I’d say they should offer him support, to indulge him more, to help me, and keep us company’.*  *C4E4*  *‘I still need to know how to manage those things, I have to pay attention. Although he also pays attention and he knows how his things are applied, the colostomy and all also, he has learnt some things’.*  *C5E5*  *‘The oxygen stops a lot, they ordered medications, we were discharged and at this time we have not gotten authorizations for any of them, not even morphine, nothing’. C1E1*  Create a Safe Environment  *They can’t see the notes . . . it’s probably wrong, because they can’t see the patient records . . . unable to solve it with any consultan - or log in, so it’s a pure stone-aged action to sit and send a test result and other stuff by fax. So that’s too bad.*  Enablers and Disablers: Communication and Coordination  *It was disorganized. There was some mis- communication. . . I ended up staying in the hospital the entire weekend unnecessarily because one of the doctors who didn’t really know me very well, said “Yeah, someone’s going to come in to see you week- end.” Well no one ever did. So they just let me go on Monday. (Patient 59M)*  *I don’t know who I was talking to, I hear one name, I hear another... And then I’d have voices on the phone and I’m, like who’s this, which one is that? Holy smokes. Come on, just stick to one or two peo- ple... because they’re all asking the same questions. (Caregiver 58M)*  *This lack of coordination led one caregiver to describe the overall transition process as “rudderless” (Caregiver 57M).* |
